# Land Finance and fiscal rules: An estimated DSGE model for Hong Kong

**DOI:** 10.1371/journal.pone.0346966

**Published:** 2026-04-15

**Authors:** Wenwen Zhang, Zongyi Liu

**Affiliations:** 1 The Center for Studies of Hong Kong, Macao and Pearl River Delta, Sun Yat-sen University, Guangzhou, China; 2 School of Economics, Nankai University, Tianjin, China; Loughborough University, UNITED KINGDOM OF GREAT BRITAIN AND NORTHERN IRELAND

## Abstract

This study systematically analyzes the choice of fiscal rules under Hong Kong’s land finance pattern and their macroeconomic implications for the first time through DSGE methods. Using quarterly data from 2000 Q1 to 2023 Q4, we employs Bayesian estimation and simulation to compare the effects of three fiscal rules: the fully exogenous rule, the output-responsive rule, and the debt-responsive rule. Results indicate that the debt-responsive rule delivers optimal outcomes in stabilizing consumption, investment, and labor markets. It effectively curbs pressures from rising government debt and property prices while enhancing fiscal sustainability. Welfare comparison results similarly confirm the superiority of the debt-responsive rule over the other two policies. Variance decomposition reveals that stamp duty shocks constitute the primary fiscal source of Hong Kong’s macroeconomic fluctuations, exerting significant impacts particularly on net exports, labor markets, and the government debt-to-revenue ratio. The conclusions of this study hold significant policy implications for Hong Kong and other economies with similar characteristics.

## 1 Introduction

As an economically developed region, Hong Kong has long been renowned for its low tax rate policy, but the fiscal revenue related to land and real estate in Hong Kong is seldom discussed. Land in Hong Kong is administered by the government and has been chronically in short supply, enabling the government to generate a significant portion of its revenue through the sale of high-priced land. Besides land transfers, the Hong Kong government directly or indirectly levies a variety of taxes on land and housing, including stamp duty related to residents, property tax, land rent and rates.

**Fig 1:**
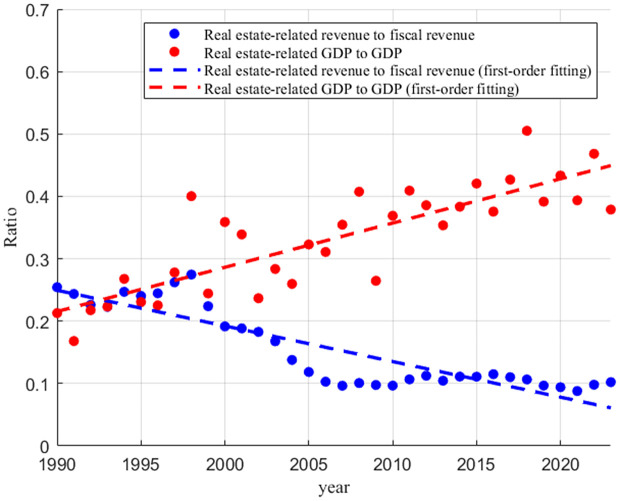
The share of Hong Kong’s real estate-related fiscal revenue and GDP within the overall total (Data source: DATA.GOV.HK).

From the late 1960s to the 1990s, Hong Kong gradually became one of the Asian Tigers with rapid economic development. Under the environment of low land supply and easing financial policy, housing prices in Hong Kong rose sharply amid volatility, the real estate economy was once in full swing. However, as shown in Fig 1, after the Asian financial crisis in 1997 and the subprime crisis in 2007, the share of output value of real estate-related sector in GDP dropped from 25.43% in 1990 to 10.22% in 2023. In contrast, the share of revenue from stamp duty, land premium, and rates increased from 21.29% to 37.89%, which indicates less contribution of real estate to economic growth and the higher dependence of government on land finance.

At the same time, over the past decade, numerous changes have occurred in fiscal policies related to the housing transaction stamp duty, land supply, and government expenditure related to the real estate industry. Take stamp duty as an illustration. The Hong Kong government introduced additional stamp duty in 2010, added the buyer’s stamp duty in 2012, increased the ad valorem stamp duty for house purchases in 2016, modified the stamp duty tax rebate policy in 2022. It can be perceived that the alterations of fiscal policy are relatively frequent and possess considerable uncertainty of land finance.

The potential risks of land finance have raised concerns about the long-term sustainability of this fiscal pattern. Pan et al. (2015) [1] pointed out that the land finance problem is one of the factors contributing to the increasing government debt burden and the weakening of the stability of the financial system, and the risk of debt-financed urbanization and economic development has already emerged under the investment-oriented economic growth model. Guo et al. (2015) [2] argued that the land market is volatile and cyclical, and the extreme dependence on land financing may amplify the economic cycle fluctuations in the aspects of government expenditure and output. Cheng et al. (2022) [3] warned that when land finance suffers a negative impact, the debt that is more flexible for the government may be affected, and it is difficult to maintain the balance of total fiscal revenue.

This paper constructs a four-sector DSGE model to discuss the land finance and fiscal rules comparison for Hong Kong. The exogenous shocks in this model include the shocks from productivity, foreign countries (consumption, prices, and interest rates), and fiscal policies (government expenditures, stamp duty, and land supply). Compared with the existing literature, the marginal contribution and innovation of this paper are mainly reflected in the following aspects:

In terms of research topics, most of the existing papers on Hong Kong economy using DSGE method focus on the stock market (Funke et al., 2011 [4]) and housing market (Funke and Paetz, 2010 [5]; Funke and Paetz, 2013 [6]; Rabanal, 2018 [7]) in the financial field, or inflation targeting in monetary systems (Lim and McNelis, 2012 [8]), linked exchange rate systems (Blagov and Funke, 2019 [9]), etc. However, so far, there are no systematic research papers on Hong Kong’s fiscal policy and land finance characteristics. For the first time, we use the DSGE method to discuss the land finance in Hong Kong, instead of focusing on the monetary and financial fields like previous studies, thus supplementing the lack of such studies.

In terms of model setting, considering the particularity of Hong Kong’s financial structure and land financial situation, we introduced fiscal variables such as stamp duty and land premium revenue, added exogenous fiscal policy shocks under different rules, and divided the firms into non-housing firms and housing firms for discussion. (such modeling methods involving real estate and land, e.g., Iacoviello and Neri, 2010 [10]; Favilukis et al., 2017 [11]; Grossman and Steger, 2017 [12]). Our model is also closely related to those of small open economies (Gali and Monacelli, 2005 [13]; Iacoviello, 2005 [14]; Monacelli, 2009 [15]). Building upon the fundamental setting of the UIP, the model further incorporates foreign consumption shocks, price shocks, and interest rate shocks, thereby better reflecting the unique characteristics of policy transmission channels in small open economies.

In terms of policy implications, existing policy research on small open economies has primarily focused on monetary policy (Benchimol, 2024 [16]; Benchimol and Bozou, 2024 [17]), whereas our study concentrates on fiscal policy, thereby filling a gap in research on small open economies. By integrating Hong Kong’s unique fiscal framework with its housing and land markets, we comprehensively assessed economic fluctuations, forecasts, and welfare losses under fully exogenous, output-responsive, and debt-responsive rules. This provides theoretical support for policymakers’ trade-offs.

Under three different fiscal rules, the impact of policy on economic fluctuations exhibits distinct variations. Through IRFs and forecasts, we find that under a fully exogenous rule, fiscal policy exerts the greatest influence on the business cycle. The rule that responds to output can smooth some fluctuations but with limited effectiveness. Only under the rule that responds to government debt can fluctuations in consumption, investment, and other areas be simultaneously mitigated while effectively alleviating debt pressures. After decomposing the variance for three shocks, namely government expenditures, stamp duties, and land supply policies, we find that stamp duties provide the strongest explanation for fluctuations in various economic variables within the business cycle. Furthermore, by comparing welfare losses under different rules, we observe that the debt-responsive rule generates the minimum welfare loss, underscoring the critical importance of prioritizing fiscal sustainability.

The rest of the paper is structured as follows. Section 2 presents the model framework. Section 3 delineates the calibration and estimation of the model. Section 4 deliberates the effects of fiscal policies under different rules. Section 5 evaluate the model performance using identification tests and Robustness tests. The last section discusses the results and concludes.

## 2 Model

The economic model formulated in this paper encompasses four sectors: households, non-housing firms, housing firms, and the government. Households consume, purchase properties, supply labor, and make investments to maximize their utility. Non-housing firms invest in capital and hire labor for production; public investment can enhance the output of such firms, while housing firms hire labor and acquire land from the government for housing construction. The government sets stamp duty rates, government expenditure, and land supply policies. Under the balanced budget constraint framework of this paper, the government’s tax revenue, land sale proceeds, and newly borrowed debt are equal to its expenditures and the repayment of principal and interest on prior-period debt.

### 2.1 Households

The households maximize the expected present value of lifetime utility, which is given by:


$${𝔼}_{0}\sum_{t=0}^{\infty }\beta^{t}(\ln C_{t}+\xi \ln H_{t}-\frac{L_{t}^{1+\varphi }}{1+\varphi })$$ (1)

where *C*_*t*_ denotes households’ consumption other than housing, *H*_*t*_ represents the housing stock purchased by households, and *L*_*t*_ indicates the labor supply. ξ measures the housing preference of households. β is the discount factor, while φ represents the Frisch elasticity of labor supply.

In the process of household investment, the adjustment cost of investment in the form of CEE (Christiano et al., 2005 [18]) is generated, which satisfies the following constraints in decision-making:


$$\begin{array}{ccc} & & P_{t}C_{t}+P_{t}I_{t}+(1+\tau_{s,t})P_{h,t}[H_{t}-(1-\delta_{h})H_{t-1}]+B_{t}+B_{f,t}\\ & & \;=W_{t}L_{t}+R_{k,t}K_{t-1}+(1+R_{t-1})B_{t-1}+(1+R_{f,t-1})\frac{B_{f,t-1}}{\Xi_{t-1}} \end{array}$$ (2)

$$K_{t}=[1-\frac{\Omega }{2}(\frac{I_{t}}{I_{t-1}}-1{)}^{2}]I_{t}+(1-\delta_{k})K_{t-1}$$ (3)

where *P*_*t*_ is the non-housing goods price so that the inflation rate is defined by $\pi_{t}=\frac{P_{t}}{P_{t-1}}$. *P*_*h*,*t*_ denotes the housing price. *I*_*t*_ is the investment amount, while *W*_*t*_ is the labor wage. *K*_*t*_ is the household capital holdings, which yield a capital return rate of *R*_*k*,*t*_. The households still hold bonds *B*_*p*,*t*_ domestically and *B*_*f*,*t*_ internationally, corresponding to the interest rates *R*_*t*_ and *R*_*f*,*t*_, separately. $\tau_{s,t}$ is the stamp duty rate based on the housing transaction amount for households. For households, stamp duty is payable only upon purchasing or transferring a residential property. This tax is levied concurrently with the transaction, with both parties bearing the tax liability. The tax base is determined by the transaction value of the property. $\delta_{h}$ and $\delta_{k}$ are the depreciation rates of houses and capital, and Ω measures the size of the adjustment cost of the investment.

Referring to Grohé and Uribe (2003) [19], we introduce the concept of debt elastic interest rate, where the domestic interest rate is defined as the sum of the foreign interest rate *R*_*f*,*t*_ and an increasing function $\Xi_{t}$. Consequently, the interest rate differential between domestic and foreign markets depends on the magnitude of foreign bond holdings *B*_*f*,*t*_ relative to the steady-state value $\overset{―}{B_{f}}$, that is:


$$\Xi_{t}=\exp[\gamma (B_{f,t}-\overset{―}{B_{f}})]$$ (4)

Households choose the contingent sequence $\{C_{t},H_{t},L_{t},K_{t},I_{t},B_{t},B_{f,t}{\}}_{0}^{\infty }$ to maximize Eq. (1) subject to Eq. (2) and Eq. (3). The first order conditions for households are:


$$U_{c,t}=P_{t}\lambda_{t}$$ (5)

$$U_{h,t}=(1+\tau_{s,t})P_{h,t}\lambda_{t}-\beta {𝔼}_{t}(1+\tau_{s,t+1})(1-\delta_{h})P_{h,t+1}\lambda_{t+1}$$ (6)

$$\lambda_{t}W_{t}=\psi L_{t}^{\varphi }$$ (7)

$$\lambda_{t}=\mu_{t}[1-\frac{\Omega }{2}(\frac{I_{t}}{I_{t-1}}-1{)}^{2}-\Omega (\frac{I_{t}}{I_{t-1}}-1)\frac{I_{t}}{I_{t-1}}]+\beta {𝔼}_{t}\mu_{t+1}\Omega (\frac{I_{t+1}}{I_{t}}-1)(\frac{I_{t+1}}{I_{t}}{)}^{2}$$ (8)

$$\lambda_{t}=\beta {𝔼}_{t}\lambda_{t+1}(1+R_{t})$$ (9)

$$\mu_{t}=\beta [R_{k,t+1}\lambda_{t}+(1-\delta_{k}){𝔼}_{t}\mu_{t+1}]$$ (10)

$$\lambda_{t}=\beta {𝔼}_{t}\lambda_{t+1}\frac{1+R_{f,t}}{\Xi_{t}}$$ (11)

where $\lambda_{t}$ and $\mu_{t}$ are Lagrange multipliers associated with two budget constraints Eq. (1) and Eq. (2). *U*_*c*,*t*_ represents the marginal utility of a unit of non-housing consumption, and *U*_*h*,*t*_ represents the marginal utility of holding an additional unit of housing. Eq. (5) and Eq. (6) are first order conditions for non-housing consumption and housing dynamics. Eq. (7) relates to household labor choice, while Eq. (8) and Eq. (9) pertain to investment and domestic bonds. Eq. (10) and Eq. (11) respectively reflect the optimal choices for capital and foreign bonds.

### 2.2 Firms

#### 2.2.1 Non-housing sector.

Suppose that the non-housing sector invests capital and employs labor to produce goods, which consists of monopolistically competitive intermediate goods producers indexed by a continuum of j∈[0,1]. The government promotes non-housing production by enhancing investment efficiency; the size of its direct or indirect effects is uniformly measured by ν. It is an important manifestation of the government expenditure multiplier effects (Wang and Wen, 2013 [20]; Guo et al., 2015 [2]). The production function is set in the form of Cobb-Douglas:


$$Y_{t}(j)=A_{t}K_{t}(j{)}^{\alpha }L_{n,t}(j{)}^{1-\alpha }G_{t}^{\nu }$$ (12)

where *Y*_*t*_(*j*) denotes the non-housing output from intermediate goods producers. Non-housing factor productivity *A*_*t*_ follows the AR(1) process $\hat{A_{t}}=\rho_{a}\hat{A_{t-1}}+\varepsilon_{a,t}$. $\hat{A_{t}}$ is the logarithmic deviation from the steady state, $\varepsilon_{a,t}\sim N(0,\sigma_{a}^{2})$. *L*_*n*,*t*_ and *K*_*t*_ are the labor and capital inputs of non-housing firms, and *G*_*t*_ represents the public expenditure of the government. We suppose that the agents of non-housing firms know the magnitude of government expenditure before each period of production decision-making. The parameter α∈(0,1) represents the output elasticity of capital, and ν∈(0,1) represents the degree to which government expenditure increases output.

The first-order conditions for maximizing profits are given by:


$$\alpha P_{t}A_{t}K_{t}(j{)}^{\alpha }L_{n,t}(j{)}^{1-\alpha }G_{t}^{\nu }=R_{k,t}K_{t}(j)$$ (13)

$$\left(1-\alpha )P_{t}A_{t}K_{t}(j{)}^{\alpha }L_{n,t}(j{)}^{1-\alpha }G_{t}^{\nu }=W_{t}L_{n,t}(j\right)$$ (14)

#### 2.2.2 Housing sector.

We assume that the government imposes the stamp duty on housing transaction amounts between housing firms and households at a tax rate of $\tau_{s,t}$. Referring to Guo et al.(2015) [2], it is assumed that the housing firms also follow the production function in the form of Cobb-Douglas, and employ labor and land to produce housing for households. Thus, the dynamic process of housing stock is:


$$H_{t}=(1-\delta_{h})H_{t-1}+V_{t}=(1-\delta_{h})H_{t-1}+Z_{t}^{\theta }L_{h,t}^{1-\theta }$$ (15)

where *L*_*h*,*t*_ and *Z*_*t*_ represent the labor and land input, respectively. The parameter θ∈(0,1) is the output elasticity of land in the housing sector.

Similarly, as the land prices *P*_*z*,*t*_ are given, the first order conditions for housing firms to maximize profits are:


$$\theta P_{h,t}[H_{t}-(1-\delta_{h})H_{t-1}]=P_{z,t}Z_{t}$$ (16)

$$(1-\theta )P_{h,t}[H_{t}-(1-\delta_{h})H_{t-1}]=W_{t}L_{h,t}$$ (17)

### 2.3 Government

Assuming the government implements a balanced budget, its revenue derives from stamp duties from households, land sale income, and borrowed debt *D*_*t*_, while expenditures stem from government spending and the repayment of prior-period debt with accrued interest. The balanced budget is given by:


$$G_{t}+(1+R_{t-1})D_{t-1}=\tau_{s}P_{h,t}[H_{t}-(1-\delta_{h})H_{t-1}]+P_{z,t}Z_{t}+D_{t}$$ (18)

In the study by Iacoviello and Neri (2010) [14], collateral constraints primarily focused on the household level and the housing market, while Liu et al. (2013) [21] incorporated land into the collateral constraints of firm debt. Drawing on the characteristics of land-based fiscal systems, we integrate land into the collateral constraints of government debt. The government’s debt ceiling is determined by the expected value of the remaining land as collateral and the LTV ratio:


$$D_{t}\leq \phi {𝔼}_{t}\frac{P_{z,t+1}(S_{t}-Z_{t})}{1+R_{t}}$$ (19)

where the supply quota *S*_*t*_ is decided by the government in each period.

The fiscal policies primarily considered in this paper focus on three aspects: government expenditures, stamp duty rates, and land supply constraints. We denote the logarithms of these policies as $\hat{{𝐗}_{t}}=(\begin{array}{c} \hat{G_{t}}\\ \hat{\tau_{s,t}}\\ \hat{S_{t}} \end{array})$, where $\overset{―}{𝐗}=(\begin{array}{c} \overset{―}{G}\\ \overset{―}{\tau_{s}}\\ \overset{―}{S} \end{array})$ are the corresponding steady-state values. For fiscal policy formulation, we consider three distinct approaches. The first is the most common specification in DSGE literature, where fiscal policy is viewed as a random exogenous shock independent of endogenous economic indicators. It is denoted as Rule 1 as follows:


$$\text{Rule 1: }\hat{{𝐗}_{t}}=\rho_{x}\hat{{𝐗}_{t-1}}+\varepsilon_{x,t}$$ (20)

Here, $\rho_{x}$ represents the first-order autoregressive coefficient of the AR(1) process, indicating the response of the current fiscal policy to the previous period. Furthermore, according to Han (2021) [22], fiscal policymaking is more purposeful than a simple AR(1) process. In the second case, for the sake of elaboration, we adopt the most common standard fiscal rule that responds to output (Guerguil et al., 2017 [23]; Combes et al., 2017 [24]), which is denoted as Rule 2 and takes the form of:


$$\text{Rule 2: }\hat{{𝐗}_{t}}=\rho_{x}\hat{{𝐗}_{t-1}}+(1-\rho_{x})\rho_{xy}\hat{{𝐘}_{t-1}}+\varepsilon_{x,t}$$ (21)

The parameter $\rho_{xy}=(\begin{array}{c} \rho_{gy}\\ \rho_{sy}\\ \rho_{zy} \end{array})$ represents the degree to which the policy responds to output. Beyond this, policy interventions may target not only output but also debt (Kliem and Kriwoluzky, 2014 [25]). In the third case, fiscal policies designed based on existing debt levels reflect the government’s concern for fiscal sustainability, which is given by:


$$\text{Rule 3: }\hat{{𝐗}_{t}}=\rho_{x}\hat{{𝐗}_{t-1}}+(1-\rho_{x})\rho_{xd}\hat{{𝐃}_{t-1}}+\varepsilon_{x,t}$$ (22)

The parameter $\rho_{xd}=(\begin{array}{c} \rho_{gd}\\ \rho_{sd}\\ \rho_{zd} \end{array})$ represents the degree to which the policy responds to existing government debt.

### 2.4 International sector

In the international sector, following Gali and Monacelli (2005) [13] and Obstfeld et al.(2010) [26], consumption consists of domestic goods *C*_*d*,*t*_ and imports *IM*_*t*_. We assume its aggregation follows a CES form:


$$C_{t}=[\chi^{\frac{1}{\eta }}C_{d,t}^{\frac{\eta -1}{\eta }}+(1-\chi {)}^{\frac{1}{\eta }}IM_{t}^{\frac{\eta -1}{\eta }}{]}^{\frac{\eta }{\eta -1}}$$ (23)

where χ indicates the allocation parameter for domestic and foreign goods, while η stands for the elasticity of substitution. Let *P*_*d*,*t*_ and *P*_*f*,*t*_ denote the prices of domestic goods and foreign goods. The budget constraint is given by:


$$P_{t}C_{t}=P_{d,t}C_{d,t}+P_{f,t}{IM}_{t}$$ (24)

Then, the optimal quantities of *C*_*d*,*t*_ and *IM*_*t*_ are obtained as follows:


$$C_{d,t}=\chi (\frac{P_{d,t}}{P_{t}}{)}^{-\eta }C_{t}$$ (25)

$$IM_{t}=(1-\chi )(\frac{P_{f,t}}{P_{t}}{)}^{-\eta }C_{t}$$ (26)

According to the symmetry, export *EX*_*t*_ equals foreign import. Similarly, the export can be derived from Eq. (23) and Eq. (24) as:


$$EX_{t}=(1-\chi_{f})(\frac{P_{f,t}}{P_{t}}{)}^{\eta_{f}}C_{f,t}$$ (27)

where *C*_*f*,*t*_ represents the foreign consumption. $\chi_{f}$ indicates the allocation parameter for goods in foreign countries, while $\eta_{f}$ stands for the foreign elasticity of substitution.

Exogenous shocks originating abroad are transmitted through price and consumption channels. Assuming *P*_*f*,*t*_, *C*_*f*,*t*_ and *R*_*f*,*t*_ follow AR(1) processes:


$$\hat{P_{f,t}}=\rho_{p_{f}}\hat{P_{f,t-1}}+\varepsilon_{p_{f},t}$$ (28)

$$\hat{C_{f,t}}=\rho_{c_{f}}\hat{C_{f,t-1}}+\varepsilon_{c_{f},t}$$ (29)

$$\hat{R_{f,t}}=\rho_{r_{f}}\hat{R_{f,t-1}}+\varepsilon_{r_{f},t}$$ (30)

where $\rho_{p_{f}}$, $\rho_{c_{f}}$, and $\rho_{r_{f}}$ are the autoregressive coefficients of foreign prices, consumption, and interest rate, $\varepsilon_{p_{f},t}\sim N(0,\sigma_{p_{f}}^{2})$, $\varepsilon_{c_{f},t}\sim N(0,\sigma_{c_{f}}^{2})$, $\varepsilon_{r_{f},t}\sim N(0,\sigma_{r_{f}}^{2})$.

Moreover, under the balance of payments, the dynamic process of foreign bonds satisfies the following condition:


$$B_{f,t}=(1+R_{f,t-1})B_{f,t-1}+\frac{P_{t}}{P_{f,t}}EX_{t}-IM_{t}$$ (31)

### 2.5 Equilibrium

The competitive equilibrium consists of the price sequence $\{R_{t},R_{k,t},R_{f,t},w_{t},\pi_{t},P_{t},P_{d,t},P_{f,t},P_{h,t},P_{z,t}{\}}_{0}^{\infty }$, the allocation sequence $\{C_{t},C_{d,t},C_{f,t},L_{t},L_{n,t},L_{h,t},V_{t},H_{t},I_{t},K_{t},Z_{t},B_{f,t},D_{t},Y_{t},IM_{t},EX_{t}{\}}_{0}^{\infty }$ and the fiscal policy sequence $\{G_{t},\tau_{s,t},LS_{t}{\}}_{0}^{\infty }$ such that (i) taking all prices and fiscal policies as given, households’ allocations maximize their utility, (ii) taking all prices and fiscal policies as given, firms’ allocations maximize their profits, and (iii) all markets clear. In addition to the first-order conditions listed above in this article, the market clearing conditions need to be satisfied in equilibrium.

The labor force provided by households is the sum of employed labor in the non-housing sector and the housing sector. The labor market clearing condition is:


$$L_{t}=\int_{0}^{1}L_{n,t}(j)dj+L_{h,t}$$ (32)

The clearing condition for the goods market is:


$$Y_{t}=C_{t}+I_{t}+G_{t}+EX_{t}-IM_{t}$$ (33)

## 3 Calibration and estimation

The parameters in the model are obtained by three methods: reference to existing literature, calibration based on real data, and Bayesian estimation. We calibrated the parameters using the long-term average economic data ratios for Hong Kong from 2000 Q1 to 2023 Q4. Specifically, $\frac{C}{Y}=0.6498$, $\frac{I}{Y}=0.2042$, $\frac{G}{Y}=0.1138$, $\frac{IM}{Y}=1.8753$, $\frac{EX}{Y}=1.9075$. Based on the above data, we calibrated households’ time discount rate to β=0.9935 since the quarterly interest rate is approximate to 0.65%, thereby ensuring our model aligns with long-term economic observations. According to the calculation of Song et al. (2011) [27], the setting of Guo et al. (2015) [2], and the database of CQER and Penn-World Table, the parameters 1−α and 1−θ, which determine the share of labor income of non-real estate firms and real estate firms, are set to 0.5 and 0.7. Simultaneously, according to Iacoviello and Neri (2010) [10], the capital depreciation rate $\delta_{k}$ and housing depreciation rate $\delta_{h}$ are set at 0.025 and 0.01, respectively.

For the parameters whose values vary greatly in different literature and are difficult to determine through calibration, this paper uses the Bayesian method to estimate the parameters. Table 1 reports the prior distribution of estimated parameters. For the reference of Guo et al. (2015) [2], the prior distribution of the parameter ν, which measures the degree to which government expenditure promotes output, is set as the Gamma distribution with a mean of 0.2 and a standard deviation of 0.1. Referring to Kliem and Kriwoluzky (2014) [25], the prior distribution of the investment adjustment cost parameter Ω is set as the Gamma distribution with a mean of 4 and a standard deviation of 0.75. Grohé and Uribe (2003) [19] found that the coefficient that interest rate risk premium exhibits the relative sensitivity to external debt λ is close to 1. We model this as a Gamma distribution with a mean of 1 and a standard deviation of 0.5. Following the study of Hong Kong by Zhao and Tang (2024) [28], the prior means of consumption allocation parameters χ is set to 0.4, and the prior mean of domestic substitution elasticity η and foreign domestic substitution elasticity are calibrated to 1. The prior mean of labor Frisch elasticity is φ=3. Referring to Kliem and Kriwoluzky (2014) [25], the prior distribution of the fiscal policy smoothing coefficient is set as the Beta distribution with a mean of 0.8 and a standard deviation of 0.1, and the prior distribution of the response coefficients of fiscal policy is set as the Normal distribution with a mean value of 0 and a standard deviation of 0.5. For the other AR(1) processes, the prior distribution of the autoregressive coefficients is set as the Beta distribution with a mean value of 0.8 and a standard deviation of 0.1, and the prior distribution of the standard deviation parameters is set as a mean value of 0.05 and a standard deviation of infinity in the Inverse Gamma distribution. Table 1 provides a detailed report of the prior distributions for all parameters to be estimated.

**Table 1:** Prior distributions of parameters.

Parameter	Description	Prior distribution
ν	Production elasticity of government expenditure	Gamma(0.2,0.1)
Ω	Investment adjustment cost coefficient	Gamma(4,0.75)
λ	Response parameter of risk premium to foreign debt	Gamma(1,0.5)
φ	Frisch labor elasticity	Gamma(3,1)
η	domestic consumption substitution elasticity	Gamma(1,0.5)
$\eta_{f}$	foreign consumption substitution elasticity	Gamma(1,0.5)
χ	consumption allocation parameter	Beta(0.4,0.1)
$\rho_{a}$	Autoregressive coefficient of productivity	Beta(0.8,0.1)
$\rho_{c_{f}}$	Autoregressive coefficient of foreign consumption	Beta(0.8,0.1)
$\rho_{p_{f}}$	Autoregressive coefficient of foreign price	Beta(0.8,0.1)
$\rho_{r_{f}}$	Autoregressive coefficient of foreign interest rate	Beta(0.8,0.1)
$\rho_{\tau_{s}}$	Stamp duty smoothing parameter	Beta(0.8,0.1)
$\rho_{g}$	Government expenditure smoothing parameter	Beta(0.8,0.1)
$\rho_{z}$	Land supply smoothing parameter	Beta(0.8, 0.1)
$\rho_{sy}$	Response parameter of stamp duty to output	Normal(0,0.5)
$\rho_{gy}$	Response parameter of government expenditure to output	Normal(0,0.5)
$\rho_{zy}$	Response parameter of land supply to output	Normal(0,0.5)
$\rho_{sd}$	Response parameter of stamp duty to debt	Normal(0,0.5)
$\rho_{gd}$	Response parameter of government expenditure to debt	Normal(0,0.5)
$\rho_{zd}$	Response parameter of land supply to debt	Normal(0,0.5)
$\sigma_{a}$	Standard deviation of productivity shock	Inverse Gamma(0.05,∞)
$\sigma_{c_{f}}$	Standard deviation of foreign consumption shock	Inverse Gamma(0.05,∞)
$\sigma_{p_{f}}$	Standard deviation of foreign price shock	Inverse Gamma(0.05,∞)
$\sigma_{r_{f}}$	Standard deviation of foreign interest rate shock	Inverse Gamma(0.05,∞)
$\sigma_{\tau_{s}}$	Standard deviation of stamp duty shock	Inverse Gamma(0.05,∞)
$\sigma_{g}$	Standard deviation of government expenditure shock	Inverse Gamma(0.05,∞)
$\sigma_{z}$	Standard deviation of land supply shock	Inverse Gamma(0.05,∞)

We incorporate as many observed variables as possible using Bayesian estimation, aligning with the number of shocks in this paper while ensuring no multicollinearity, thereby guaranteeing the accuracy of identification. Specifically, we selected seven sets of quarterly-frequency variables from Hong Kong’s economic fundamentals between the first quarter of 2000 and the fourth quarter of 2023. These variables are: output, consumption, imports, government expenditure, the housing price index, the amount of housing transaction contracts, and employment. Macroeconomic fundamentals, including data on output, consumption, imports, government expenditure, and employment, are sourced from the Hong Kong Census and Statistics Department (DATA.GOV.HK). Property price indices and the amount of housing transaction contracts are sourced from the Rating and Valuation Department (rvd.gov.hk) and the Land Registry (landreg.gov.hk), respectively. For detailed data information, see S1 Table. All data have been detrended to maintain the stationarity of the time series. Following the critique by Hamilton (2018) [29], we adopt a first-order detrending method rather than the HP filter. The mapping relationship between observed variables and endogenous variables $\left\{Y_{obs,t},C_{obs,t},IM_{obs,t},G_{obs,t},P_{h,obs,t},\Delta H_{obs,t},L_{obs,t}\right\}$ in the model proceeds as follows:


$$\left[\begin{array}{c} \log(Y_{obs,t})\\ \log(C_{obs,t})\\ \log(IM_{obs,t})\\ \log(G_{obs,t})\\ \log(P_{h,obs,t})\\ \log(\Delta H_{obs,t})\\ \log(L_{obs,t}) \end{array}]=[\begin{array}{c} \log(\overset{―}{Y})+\hat{Y_{t}}\\ \log(\overset{―}{C})+\hat{C_{t}}\\ \log(\overset{―}{IM})+\hat{IM_{t}}\\ \log(\overset{―}{G})+\hat{G_{t}}\\ \log(\overset{―}{P_{h}})+\hat{P_{h,t}}\\ \log(\overset{―}{V})+\hat{V_{t}}\\ \log(\overset{―}{L})+\hat{L_{t}} \end{array}\right]$$ (34)

where parameters $\overset{―}{Y},\overset{―}{C},\overset{―}{IM},\overset{―}{G},\overset{―}{P_{h}},\overset{―}{V},\overset{―}{L}$ represent the steady-state values of output, consumption, imports, government expenditures, housing price, housing stock increment, and employment. Table 2 presents the posterior distribution estimates of the parameters under the three different rules described above.

**Table 2:** Posterior distributions of parameters.

Parameter	Posterior distribution					
	Rule 1		Rule 2		Rule 3	
	Mean	90% Interval	Mean	90% Interval	Mean	90% Interval
ν	0.0817	[0.0196,0.1389]	0.0436	[0.0094,0.0782]	0.0900	[0.0252,0.1484]
Ω	4.4021	[3.1795,5.8142]	4.6723	[3.2086,6.1493]	4.5398	[3.1668,5.8979]
λ	3.7240	[2.5024,4.8719]	2.6040	[1.5332,3.6252]	0.2965	[0.0988,0.4759]
φ	3.6231	[2.9767,4.2145]	3.4345	[2.8295,4.0904]	3.6639	[3.0274,4.3515]
η	2.8714	[2.1102,3.7902]	5.0490	[3.6004,6.6242]	2.9493	[2.0132,3.8396]
$\eta_{f}$	1.9537	[0.9830,3.1355]	3.7640	[1.9438,5.2736]	3.3498	[1.9538,4.5656]
χ	0.4127	[0.2583,0.5727]	0.3362	[0.1968,0.4783]	0.3679	[0.2186,0.5165]
$\rho_{a}$	0.8591	[0.8098,0.9077]	0.7929	[0.7037,0.8813]	0.9235	[0.8962,0.9530]
$\rho_{c_{f}}$	0.6837	[0.6171,0.7464]	0.7880	[0.7190,0.8646]	0.8474	[0.7865,0.9071]
$\rho_{p_{f}}$	0.9594	[0.9510,0.9673]	0.9646	[0.9558,0.9751]	0.9881	[0.9864,0.9899]
$\rho_{r_{f}}$	0.9227	[0.8759,0.9733]	0.8476	[0.7497,0.9643]	0.6933	[0.5971,0.7826]
$\rho_{\tau_{s}}$	0.7871	[0.7617,0.8093]	0.5758	[0.5072,0.6465]	0.6003	[0.5632,0.6362]
$\rho_{g}$	0.5772	[0.4509,0.7008]	0.6594	[0.4947,0.8428]	0.5298	[0.4121,0.6578]
$\rho_{z}$	0.9812	[0.9678,0.9961]	0.9838	[0.9717,0.9956]	0.9756	[0.9600,0.9924]
$\rho_{sy}$	–	–	0.6538	[0.4733,0.8673]	–	–
$\rho_{gy}$	–	–	−0.5457	[-1.1321,0.0310]	–	–
$\rho_{zy}$	–	–	0.1473	[-0.8100,1.0522]	–	–
$\rho_{sd}$	–	–	–	–	2.3700	[1.8102,2.8582]
$\rho_{gd}$	–	–	–	–	−0.0293	[-0.2970,0.2673]
$\rho_{zd}$	–	–	–	–	0.2434	[-0.6698,1.0801]
$\sigma_{a}$	0.0572	[0.0504,0.0639]	0.0560	[0.0485,0.0630]	0.0577	[0.0505,0.0646]
$\sigma_{c_{f}}$	0.1372	[0.1084,0.1654]	0.1379	[0.1074,0.1706]	0.1625	[0.1208,0.2064]
$\sigma_{p_{f}}$	0.3207	[0.2827,0.3580]	0.3145	[0.2801,0.3518]	0.3223	[0.2843,0.3571]
$\sigma_{r_{f}}$	0.0113	[0.0087,0.0136]	0.0133	[0.0094,0.0166]	0.0134	[0.0095,0.0169]
$\sigma_{\tau_{s}}$	0.3137	[0.2740,0.3491]	0.3678	[0.3204,0.4159]	0.3677	[0.3224,0.4151]
$\sigma_{g}$	0.0590	[0.0516,0.0663]	0.0622	[0.0537,0.0711]	0.0589	[0.0512,0.0659]
$\sigma_{z}$	0.0566	[0.0429,0.0700]	0.0398	[0.0300,0.0475]	0.0994	[0.0841,0.1155]

## 4 Results

### 4.1 Impulse responses

This section builds upon the four-sector model constructed earlier to conduct impulse response analysis under three fiscal rules (Rule 1: fully exogenous rule; Rule 2: output-responsive rule; Rule 3: debt-responsive rule). It examines the dynamic effects of the government expenditure shock, stamp duty shock, and land supply shock on key variables of the Hong Kong economy. All impulse responses are simulated based on the posterior parameter means obtained through Bayesian estimation.

**Fig 2:**
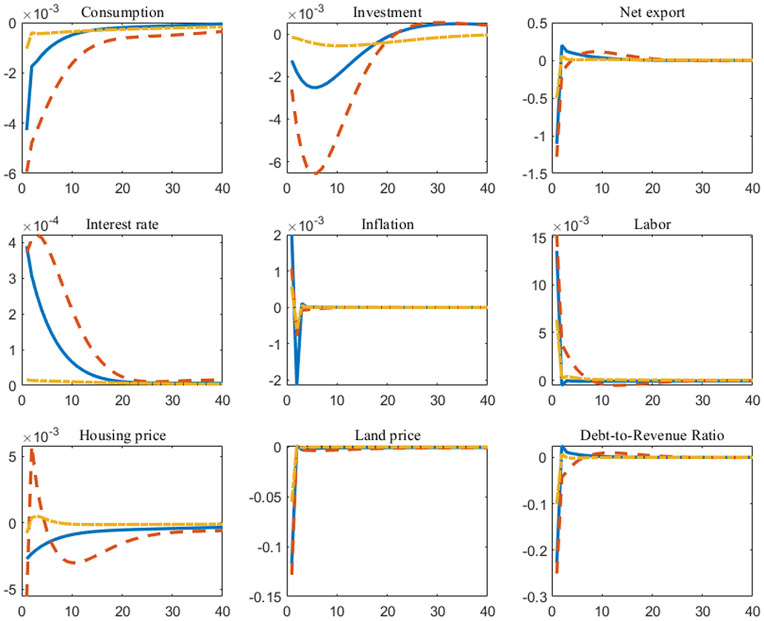
Impulse responses of government expenditure shock under different fiscal rules (*The horizontal axis in the figure represents the number of periods following the shock, while the vertical axis indicates the deviation of the variable relative to the steady state. The solid line is the case of Rule 1, the dashed line is the case of Rule 2, and the dash-dot line is the case of Rule 3*).

We find that under each rule, government expenditure exhibits the crowding-out effect to some extent, as shown in Fig 2. Under balanced budget rules, the government incurs debt to cover deficits, which primarily drives up market interest rates. Consequently, household investment declines due to high interest rates, and consumption shrinks as investment income decreases. The crowding-out effect is most pronounced under Rule 1; conversely, under Rule 3, the government will tighten spending more rapidly after shocks to maintain debt sustainability, interest rate fluctuations are relatively stable, and government expenditures exert less crowding-out pressure on consumption and investment. Meanwhile, due to the temporary rise in domestic inflation, imports increased while exports decreased in the international sector, causing net exports to follow a downward trend in the short term. In addition, government expenditures spur short-term growth in employment. This is attributable both to the stimulus of government expenditures on production and to households undertaking additional work to compensate for losses in investment income. We also note that the impact of government expenditures on housing price fluctuations varies distinctly under different policy rules. Under Rule 1 and Rule 2, fiscal rules do not prioritize fiscal sustainability. Consequently, increased government expenditures lead to heightened debt repayment pressures and potential deficit risks. Since debt levels are tied to the value of mortgaged land, expectations for the land and housing markets decline. Under Rule 3, however, government expenditures are adjusted in a timely manner based on current debt levels, thereby playing a greater role in stimulating the housing market.

**Fig 3:**
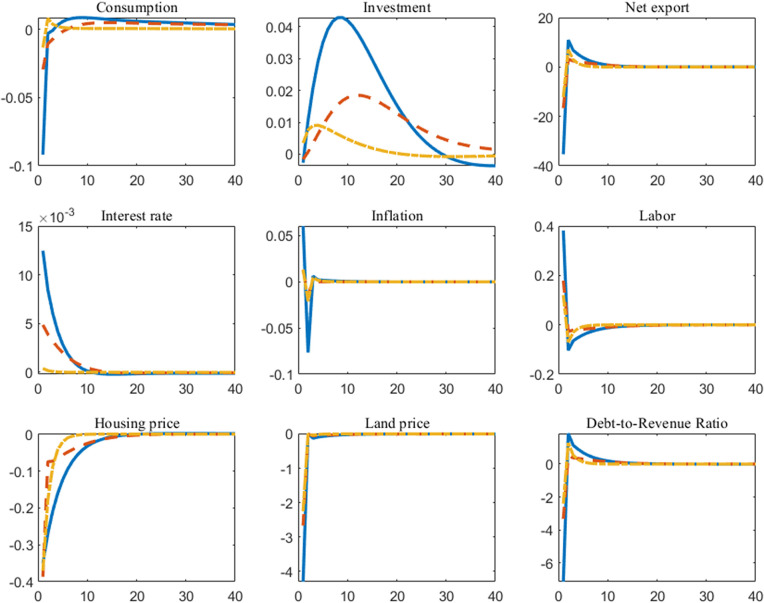
Impulse responses of stamp duty shock under different fiscal rules (*The horizontal axis in the figure represents the number of periods following the shock, while the vertical axis indicates the deviation of the variable relative to the steady state. The solid line is the case of Rule 1, the dashed line is the case of Rule 2, and the dash-dot line is the case of Rule 3*).

Stamp duty is another crucial component of the fiscal system. In Fig 3, a positive stamp duty shock demonstrates a dampening effect on consumption and a stimulating effect on investment. The underlying logic is straightforward: higher stamp duty directly increases the cost of home purchases for households, thereby reducing the disposable income available for consumption. Simultaneously, a less heated housing market encourages investors to channel more capital into the real economy, ultimately elevating overall investment levels in the macroeconomy. Rules 2 and 3 enable stamp duty policy to respond to macroeconomic conditions and debt levels, thereby smoothing fluctuations and stabilizing consumption and investment dynamics. We further observe that regardless of the rule applied, the impact of stamp duty, a tax associated with housing transactions, suppresses housing prices, which aligns with existing empirical findings (Besley et al., 2014 [30]; Kopczuk and Munroe, 2015 [31]; Best and Kleven, 2018 [32]). Under Rule 3, the government determines stamp duty rates based on debt levels relative to housing and land prices, thereby minimizing the decline in housing prices. For the land market, the increase in stamp duty reduces housing demand. This shrinking demand further translates into a decrease in land requirements for housing construction firms, leading to a corresponding decline in land prices. Subsequently, the contraction of the land market also triggers a downsizing of government debt, thereby reducing the debt-to-revenue ratio.

**Fig 4:**
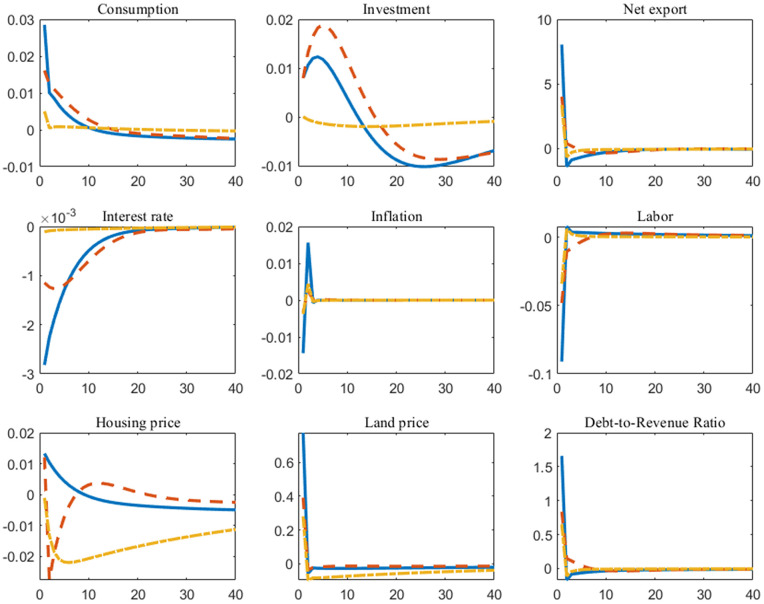
Impulse responses of land supply shock under different fiscal rules (*The horizontal axis in the figure represents the number of periods following the shock, while the vertical axis indicates the deviation of the variable relative to the steady state. The solid line is the case of Rule 1, the dashed line is the case of Rule 2, and the dash-dot line is the case of Rule 3*).

We now turn to the implications of government land supply policies. In fact, positive land supply shocks primarily manifest as a demand-pulling effect. This manifests as increased household consumption and a temporary rise in investment levels driven by falling interest rates. Compared to Rule 1 and Rule 2, implementing land supply policies under Rule 3 exerts a gentler impact on economic fundamentals. As shown in Fig 4, the IRFs for consumption, investment, and interest rates under Rule 3 exhibit relatively flatter trajectories, further underscoring Rule 3’s policy orientation toward stabilizing fluctuations. Furthermore, the loosened land supply eases constraints on land use for construction by housing enterprises, enabling firms to expand their capacity and thereby employ more labor. Under Rule 1 and Rule 2, the land supply shock conveys the signal of economic expansion, fostering positive future expectations in the market and consequently driving up housing prices and land prices. Under Rule 3, however, the land supply shock is translated into a signal of fiscal deficit pressure. Since land transactions are directly linked to fiscal debt under the established rules, this instead fosters negative expectations in the housing market, thus depressing prices. This counterintuitive outcome precisely highlights the peculiarity of the “land finance” development pattern. Moreover, raising the land supply cap also implies an increase in the debt ceiling determined by the collateral value of surplus land. As the government further expands its debt, the debt-to-revenue ratio rises, and fiscal sustainability weakens.

### 4.2 Variance decomposition

We adopt variance decomposition to analyze the contribution of different fiscal shocks to the volatility of essential economic components, thereby identifying the sources of shocks affecting Hong Kong’s economic fluctuations. Table 3 presents the percentage of theoretical variance contributed by the government expenditure shock ($\sigma_{g}$), stamp duty shock ($\sigma_{\tau_{s}}$), and land supply shock ($\sigma_{z}$) to the movements of key macroeconomic variables under three fiscal rule scenarios.

**Table 3:** Variance decomposition of fiscal shocks in percent.

Variable/Shock	Posterior distribution								
	Rule 1			Rule 2			Rule 3		
	$\sigma_{g}$	$\sigma_{\tau_{s}}$	$\sigma_{z}$	$\sigma_{g}$	$\sigma_{\tau_{s}}$	$\sigma_{z}$	$\sigma_{g}$	$\sigma_{\tau_{s}}$	$\sigma_{z}$
Consumption	0.0182	6.3857	0.9755	0.0224	0.3997	0.2968	0.0002	0.0117	0.0019
Investment	0.0019	0.6280	0.1153	0.0068	0.1231	0.1336	0.0000	0.0026	0.0006
Net export	0.0383	45.6982	2.2378	0.2562	49.5693	2.9444	0.0321	25.8255	1.5273
Interest rate	0.0162	11.8062	0.9942	0.0763	4.5341	0.9772	0.0004	0.0251	0.0113
Inflation	0.0098	11.3484	0.5563	0.0016	0.3427	0.0184	0.0007	0.6093	0.0328
Labor	0.0479	46.1490	2.4550	0.2846	44.0080	3.6392	0.0710	36.2238	2.1771
Housing price	0.0013	10.8974	0.0975	0.0064	7.5101	0.1282	0.0000	2.2667	0.1678
Land price	0.0334	47.4922	1.7207	0.1073	53.2935	1.3841	0.0214	36.1089	1.8485
Debt-to-revenue ratio	0.0408	47.6987	2.4038	0.2672	55.0455	3.7034	0.0754	58.1534	3.3933

Overall, stamp duty shocks appear to be the primary fiscal contributor to macroeconomic volatility in Hong Kong. Under all three rules, their contribution to fluctuations in variables such as the net export, labor, land price, and debt-to-revenue ratio remains significant and consistently high. For instance, under Rule 1, the stamp duty shock accounted for 45.70% fluctuations of net export, 46.15% of the labor, and 47.70% of the debt-to-revenue ratio. This finding underscores that tax policies closely tied to housing transactions possess powerful spillover effects – not only directly impacting the housing market but also broadly affecting the balance of payments, the labor market, and the sustainability of the government finance.

The contribution of land supply shocks exhibits pronounced rule dependency. Under Rules 1 and 2, their impact on fundamentals such as consumption, investment, and interest rates remains relatively moderate. Under Rule 3, the contribution of land supply shocks to land price and debt-to-revenue ratio fluctuations rises to 1.85% and 3.39%. This indicates that when fiscal rules prioritize debt sustainability, the link between land supply policies and government indebtedness is significantly strengthened. Changes in land supply directly impact government borrowing capacity and debt burdens through the collateral value channel, therefore becoming a significant source of fiscal volatility.

In contrast, the contribution of the government expenditure shock is relatively mild under all three rules, particularly under Rule 3. Its explanatory power for fluctuations in core macroeconomic variables such as consumption and investment generally falls below 0.1%. This reflects that, within Hong Kong’s land finance framework, fiscal expenditures are not the primary drivers of economic fluctuations. When fiscal rules incorporate feedback mechanisms for debt (Rules 3), government expenditures tend to function more as “automatic stabilizers” or “debt smoothers,” and their own characteristics as independent shock sources are weakened.

The variance decomposition results above indicate that Hong Kong’s macroeconomic fluctuations are closely linked to land-related fiscal instruments, particularly stamp duty. While a simple output-responsive fiscal rule (Rule 2) can moderate some fluctuations, only by explicitly anchoring the fiscal rule to debt levels (Rule 3) can the transmission of various fiscal shocks be systematically reduced. This provides a more binding policy framework to mitigate reliance on the “land finance” pattern which depends on stamp duty and land sale revenues, thereby enhancing fiscal sustainability.

### 4.3 Forecasts

**Fig 5:**
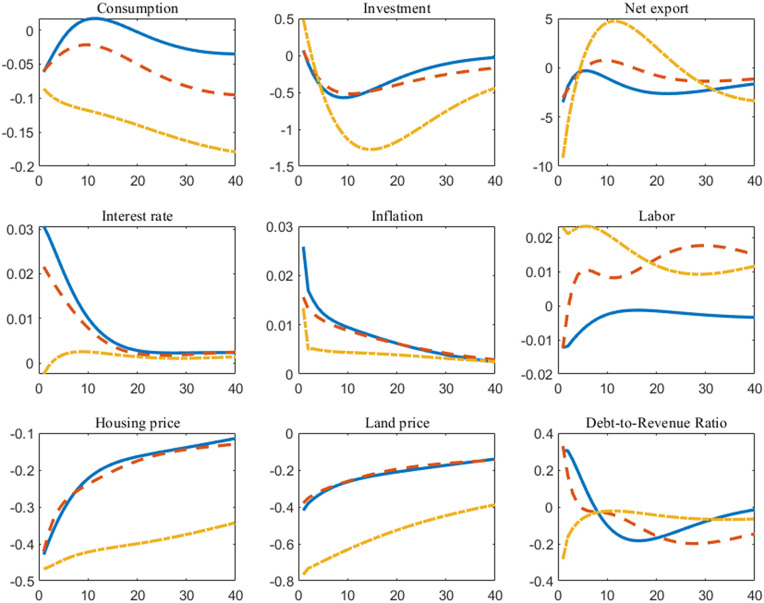
Forecasts from the posterior mean under different fiscal rules (*The horizontal axis in the figure represents the number of periods following the shock, while the vertical axis indicates the deviation of the variable relative to the steady state. The solid line is the case of Rule 1, the dashed line is the case of Rule 2, and the dash-dot line is the case of Rule 3*).

This section uses the posterior parameter means obtained from Bayesian estimation (see Table 3) to forecast the equilibrium paths of the model under three different fiscal rules. The projections simulate the percentage deviation paths of key macroeconomic variables relative to their steady-state values over the next 40 quarters, with results presented in Fig 5. The forecast path begins with the final observed value of the sample, namely Q4 2023. We employ a mean forecast, accounting solely for parameter uncertainty rather than uncertainty surrounding future shocks. The impact of shocks is neutralized by setting them to zero.

By and large, Rule 3 exhibits the most pronounced stabilizing effect. Under this rule, the forecast paths of actual variables such as consumption and investment show the smallest fluctuations and converge more rapidly toward steady states. This indicates that fiscal rules targeting government debt levels directly can effectively reduce policy-induced uncertainty by constraining government expenditures and land finance practices. By providing the private sector with more stable long-term expectations, such rules help curb excessive volatility in the economy.

Specifically, we have three key findings:

First, under Rule 1, the projected paths of output and consumption exhibit persistent cyclical fluctuations. Rule 2 partially smooths these fluctuations by linking fiscal policy to the output gap, though its effect diminishes after the medium term. In contrast, Rule 3 manages to suppress fluctuations in consumption, interest rates, and inflation within an extremely narrow range, resulting in nearly smooth paths. The underlying mechanism is that under Rule 3, the government’s adjustments to stabilize debt automatically offset other shocks in the economy, thereby maintaining intrinsic stability.

Second, regarding housing and land prices, the forecast results further highlight the divergence in policy outcomes under the land finance pattern. Under Rules 1 and 2, the forecast paths for housing and land prices exhibit sustained moderate upward pressure, paralleling the rise in government debt levels. Under Rule 3, however, as policies respond to debt accumulation, fiscal policy becomes more effective in monitoring debt dynamics, while the forecast paths for housing and land prices stabilize.

Third, the forecast paths for the government debt-to-revenue ratio show stark differences. Under Rules 1 and 2, this ratio exhibits a long-term upward trend, which portends persistent pressure on fiscal sustainability. Meanwhile, under Rule 3, the debt-to-revenue ratio is strictly anchored and stabilized near a steady-state level. This contrast strongly indicates that without explicitly linking fiscal rules to debt measures, Hong Kong’s current land finance pattern will drive continuous increases in government leverage, accumulating long-term risks. Conversely, debt-responsive rules can effectively curb this trend and provide safeguards for fiscal sustainability.

### 4.4 Welfare comparison

In the preceding discussion, we conducted only a formal qualitative analysis of the ranking of the three rules from different perspectives. To enable the rules to more intuitively reflect their overall effects quantitatively, we further calculate welfare losses using the methods of Woodford (2003) [33], Gali and Monacelli (2005) [13], and Gali and Monacelli (2008) [34], thereby illustrating the economy’s deviation from an efficient equilibrium. Specifically, we define the logarithmic deviation of variables relative to steady state as $\hat{C_{t}}=\ln(\frac{C_{t}}{\overset{―}{C}})$ and $\hat{H_{t}}=\ln(\frac{H_{t}}{\overset{―}{H}})$ $\hat{L_{t}}=\ln(\frac{L_{t}}{\overset{―}{L}})$. We can rewrite the single-period utility corresponding to Eq. (1) as:


$$U_{t}=\hat{C_{t}}+\ln\overset{―}{C}+\xi (\hat{H_{t}}+\ln\overset{―}{H})-\frac{(\overset{―}{L}e^{\hat{L_{t}}}{)}^{1+\varphi }}{1+\varphi }$$ (35)

Perform a second-order Taylor expansion of *L*_*t*_ at $\overset{―}{L}$ to obtain:


$$\begin{array}{cc} U_{t} & =\hat{C_{t}}+\ln\overset{―}{C}+\xi (\hat{H_{t}}+\ln\overset{―}{H})-[\frac{{\overset{―}{L}}^{1+\varphi }}{1+\varphi }+{\overset{―}{L}}^{1+\varphi }\hat{L_{t}}+\frac{1}{2}(1+\varphi ){\overset{―}{L}}^{1+\varphi }{\hat{L_{t}}}^{2}]\\ & =\overset{―}{U}+\hat{C_{t}}+\xi \hat{H_{t}}-{\overset{―}{L}}^{1+\varphi }\hat{L_{t}}-\frac{1}{2}(1+\varphi ){\overset{―}{L}}^{1+\varphi }{\hat{L_{t}}}^{2} \end{array}$$ (36)

Since all variables return to their long-term steady state, $𝔼[\hat{C_{t}}]=𝔼[\hat{H_{t}}]=𝔼[\hat{L_{t}}]=0$. Therefore, the welfare loss function Υ is ultimately expressed as:


$$\Upsilon ={𝔼}_{0}\sum_{t=0}^{\infty }\beta^{t}|U_{t}-\overset{―}{U}|={𝔼}_{0}\sum_{t=0}^{\infty }\beta^{t}[\frac{1}{2}(1+\varphi ){\overset{―}{L}}^{1+\varphi }{\hat{L_{t}}}^{2}]=\frac{1}{2}(1+\varphi ){\overset{―}{L}}^{1+\varphi }Var[\hat{L_{t}}]$$ (37)

The calculation results show significant differences in welfare losses under the three rules: Rule 1 incurs the highest welfare loss, reaching 3.36%. This indicates that when fiscal policy operates entirely independently of endogenous economic indicators, the inherent uncertainty of the policy amplifies economic fluctuations,especially in the labor market, which leads to substantial welfare losses. Under Rule 2, the welfare loss decreased significantly to 1.75%. This demonstrates that linking fiscal policy to the output gap can function as an automatic stabilizer, which smooths economic cycles, mitigates excessive fluctuations in the labor market, and thereby enhances welfare. Rule 3 yields the lowest welfare loss at only 1.02%. This demonstrates that fiscal rules responsive to government debt are most effective in stabilizing the economy and reducing volatility. By monitoring government spending and land-based fiscal practices, Rule 3 provides the private sector with more stable long-term expectations, thereby significantly reducing fluctuations in the labor market and the aggregate economy.

## 5 Model performance validation

### 5.1 Identification tests

To determine whether all parameters to be estimated are effectively identified, we first conducted identification tests on all parameters, following the methods of Iskrev (2010) [35], Komunjer and Ng (2011) [36], and Qu and Tkachenko (2012) [37]. The results show that under three distinct levels of testing, all parameters are identified in the Jacobian of the first two moments, the steady-state and minimal system, and the Jacobian of mean and spectrum. Then, by examining the overlap plots of prior-posterior distributions for each parameter under three different rules, we observe that the posterior distribution of each parameter shares overlapping regions with its prior distribution but does not fully coincide with it. This indicates that the posterior distribution can adjust within a given interval during the estimation process, which validates the reasonableness of prior distribution selection and the identifiability of parameters to some extent. S2 Fig details the results of the identification tests.

### 5.2 Robustness tests

To verify the consistency of the preceding results, we conducted robustness tests in two aspects, following the approach of Smets and Wouters (2007) [38]. Initially, we examined the sensitivity of the prior distribution. Specifically, we tested the sensitivity of the estimation results to prior assumptions by relaxing the standard deviation of the prior distribution to 1.1 times its original value. Re-estimation revealed that even with a more lenient standard deviation, the parameter estimates remained highly similar to the original results. This indicates that our parameter estimates exhibit no significant sensitivity to the chosen prior distribution range. Subsequently, we also take note of the critique by Ramey and Zubairy (2018) [39], carefully considering the possibility of divergent outcomes under different initial economic conditions when analyzing the economic impacts of fiscal policies. Thus, we further examined the stability of the estimates across different sample sizes. Considering the substantial impact of the SARS pandemic on the Hong Kong economy, we re-estimated the model using a subsample from 2004 onward. We find that after reducing the sample size by one seventh, the estimation results remain similar to the original estimates, indicating robustness across sample lengths. Detailed estimated results from robustness tests are presented in S3 Table.

## 6 Conclusion and discussion

This paper constructs a four-sector DSGE model encompassing households, non-housing firms, housing firms, and the government. It provides the first systematic analysis of fiscal rule choices and their macroeconomic implications under Hong Kong’s land finance pattern. Conducting Bayesian estimation and simulation using quarterly data from Hong Kong covering 2000 Q1 to 2023 Q4 under three distinct fiscal policy rules, the study arrives at the following key conclusions:

First, the design of fiscal rules is crucial for regulating land finance risks and stabilizing the macroeconomy. Research indicates that fiscal rules that are entirely exogenous (Rule 1) or respond solely to output gaps (Rule 2) may stimulate the economy in the short term but exacerbate economic volatility. They also lead to sustained upward trends in government debt and housing prices over the medium to long term, accumulating systemic risks. In contrast, the fiscal rule that responds to government debt levels (Rule 3) demonstrates significant superiority: it not only most effectively dampens fluctuations in consumption, investment, and labor markets but also stabilizes expectations for housing and land prices by constraining debt expansion backed by land collateral. This fundamentally enhances fiscal sustainability.

Second, stamp duty serves as a pivotal fiscal instrument influencing Hong Kong’s economic fluctuations. Variance decomposition results indicate that under any regulatory framework, stamp duty shocks exhibit strong explanatory power over fluctuations in net exports, the labor market, and the government debt-to-revenue ratio. This underscores that in economies heavily reliant on land finance, tax policies closely tied to housing transactions generate broad and potent spillover effects.

Third, the “land finance” pattern inherently involves a trade-off between growth and stability. Both impulse responses and forecast simulations indicate that policies aimed at reducing reliance on land finance (such as Rule 3), while enhancing long-term fiscal sustainability, may necessitate enduring short-term economic slowdown pressures during the transition period. This finding holds significant policy implications for Hong Kong and other economies with similar characteristics: Fiscal rules focused solely on economic growth targets may reinforce dependence on land finance. Transitioning to a fiscal rule framework centered on debt sustainability represents the fundamental path to resolving the fiscal dilemma and achieving long-term stable development.

We also note that Hong Kong has formally abolished all demand-side residential stamp duty measures effective February 28, 2024, including the Special Stamp Duty, Buyer’s Stamp Duty, and New Residential Properties Stamp Duty (ird.gov.hk). This institutional change alters the stamp duty channel, which served as a key driver of dynamic shifts and fiscal revenue in our research. Once relevant statistical data become available, we will explore the impact of this policy adjustment on Hong Kong’s land finance pattern as a future research extension.

## Supporting information

S1 Table Data used in this paper. (XLSX)

S2 Fig Supplementary figures for identification tests. (DOCX)

S3 Table Supplementary tables for robustness tests. (DOCX)
