# Supplementary material for: Land Finance and fiscal rules: An estimated DSGE model for Hong Kong
Source: PLoS One. 2026 Apr 15;21(4):e0346966. doi: 10.1371/journal.pone.0346966 (PMC13082663; doi:10.1371/journal.pone.0346966)
Supplement: S2 Fig — (DOCX) [file pone.0346966.s002.docx]

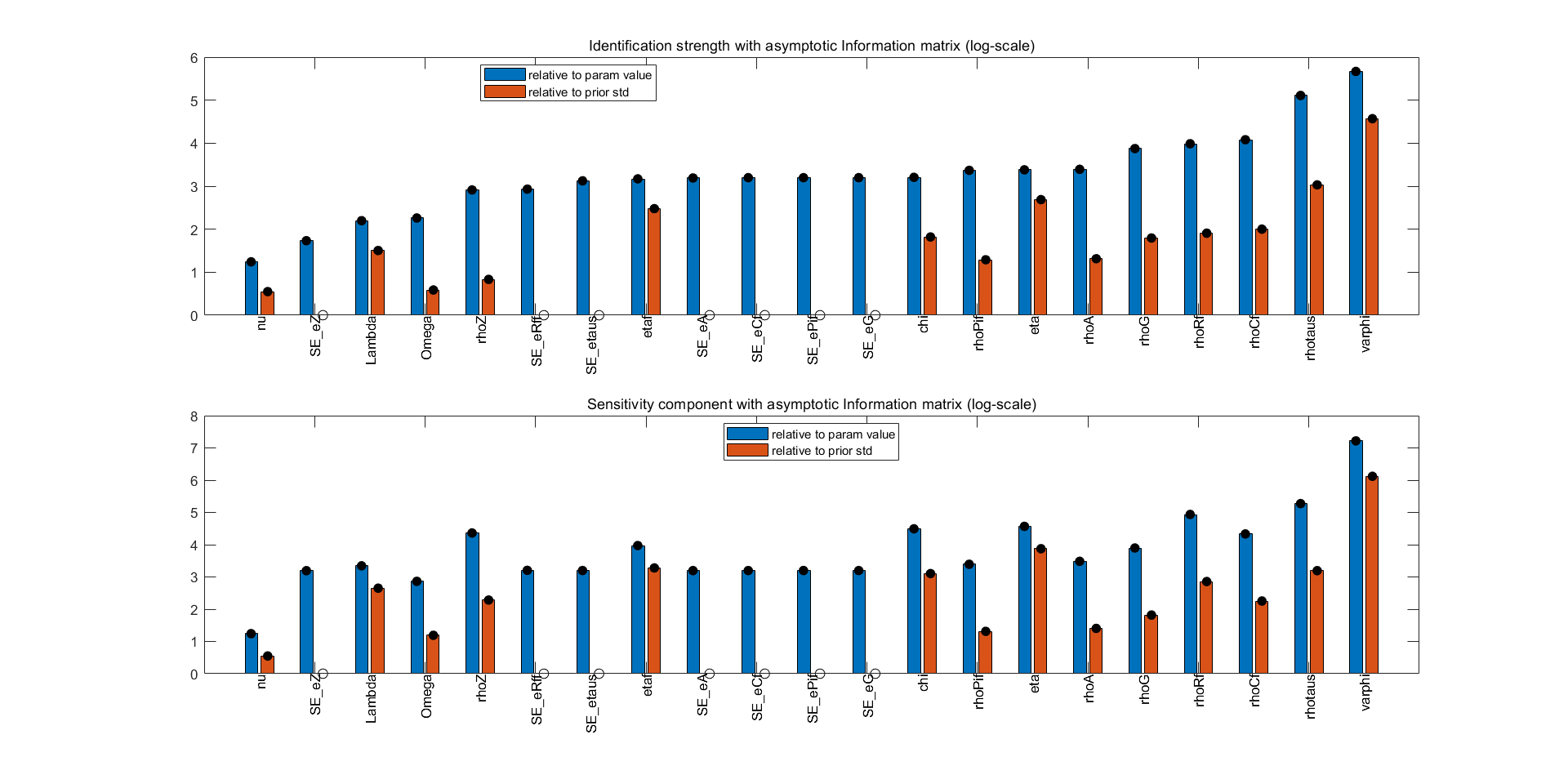


Fig 1. Identification test result under Rule 1.


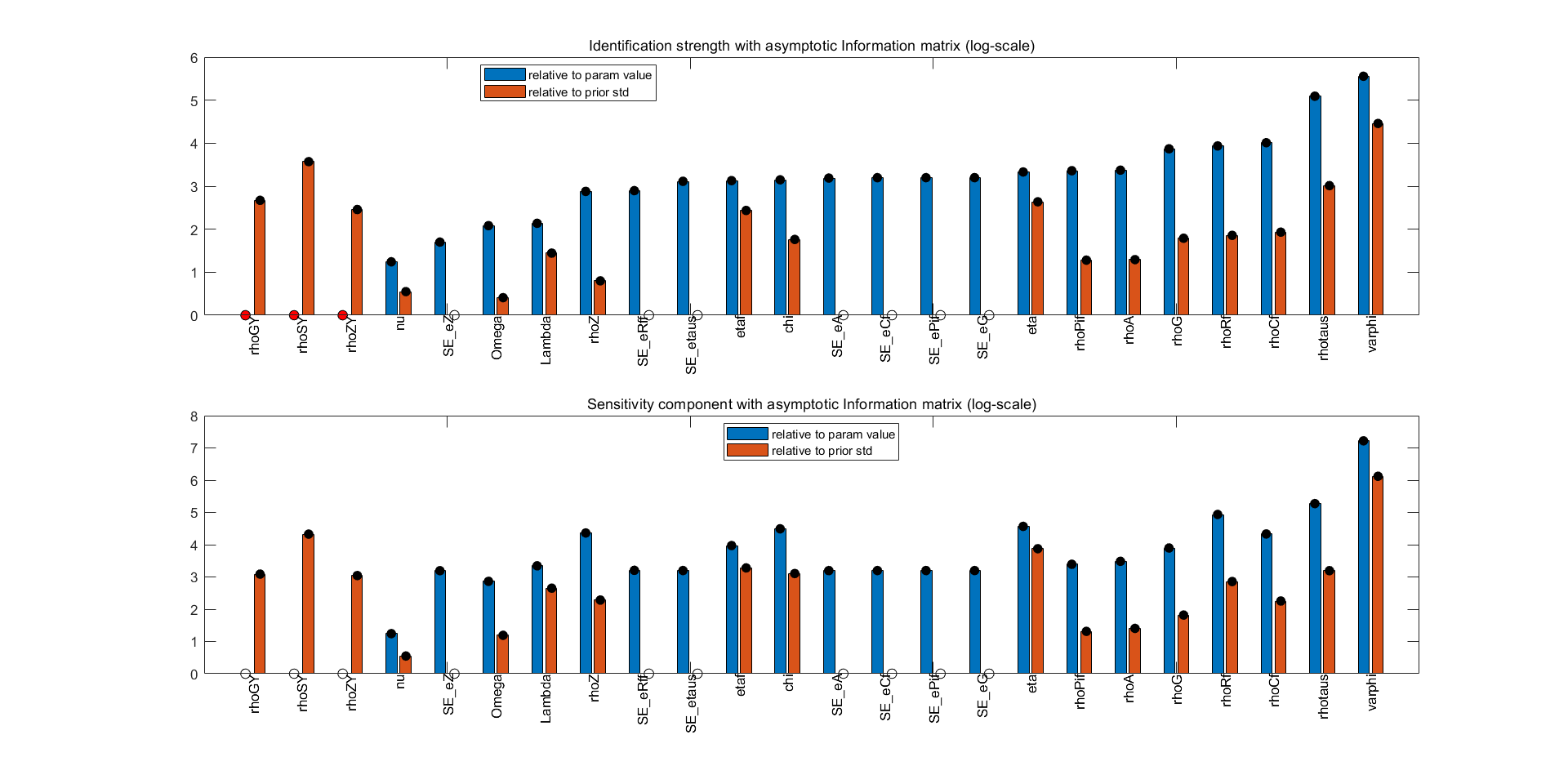


Fig 2. Identification test result under Rule 2.


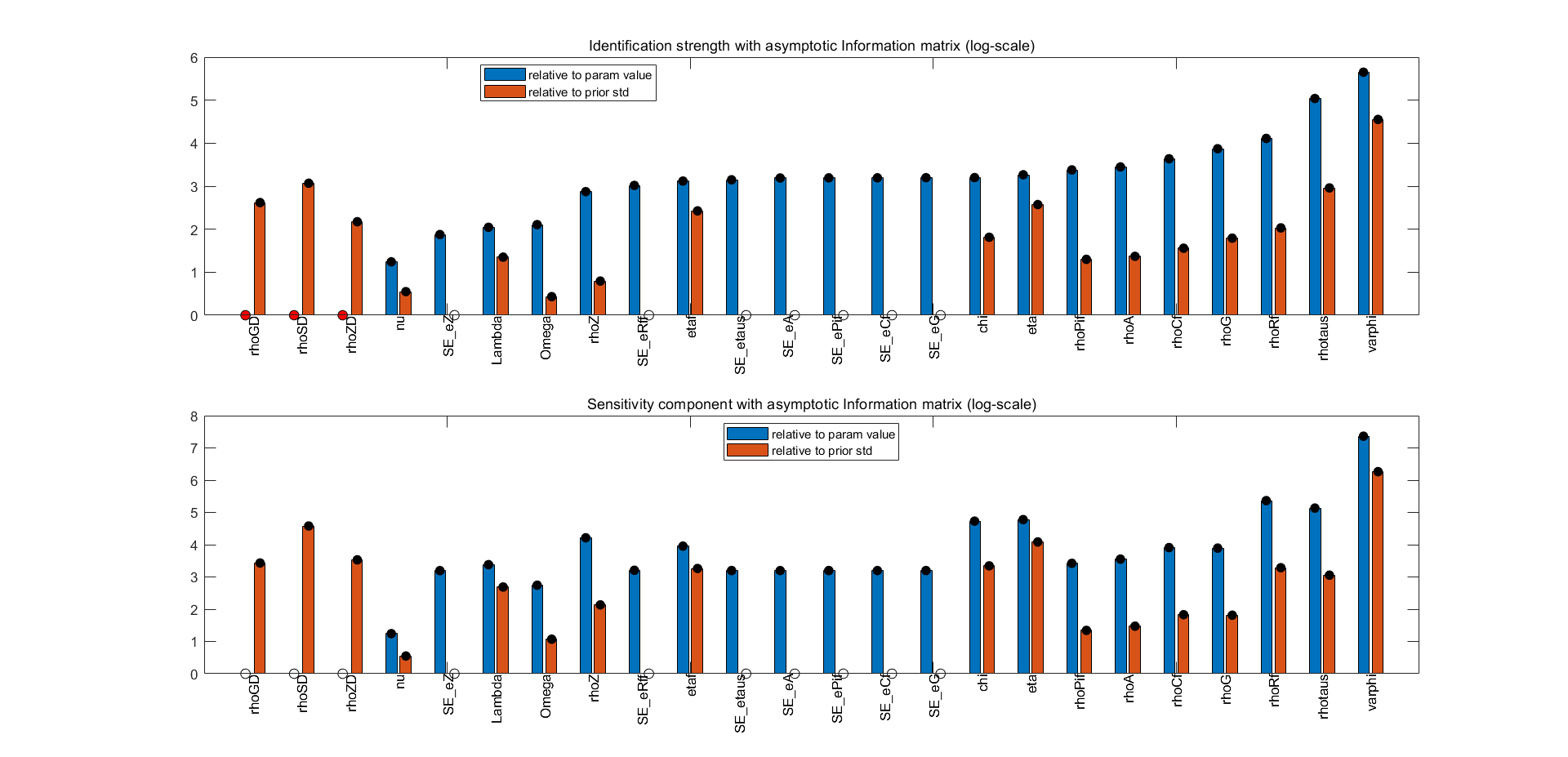


Fig 3. Identification test result under Rule 3.


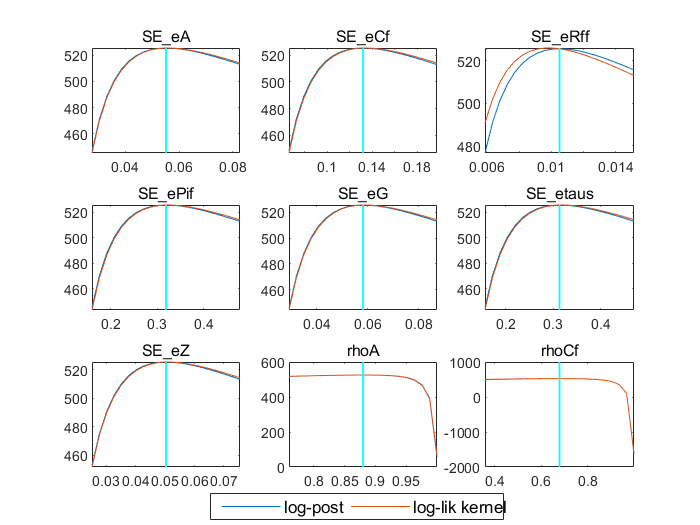

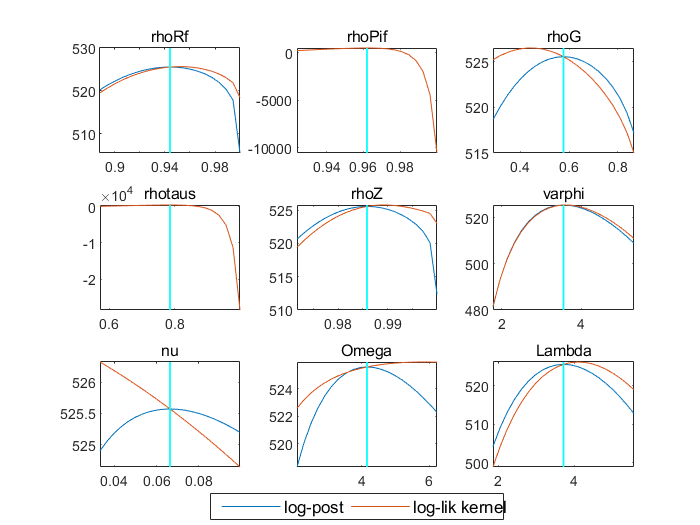

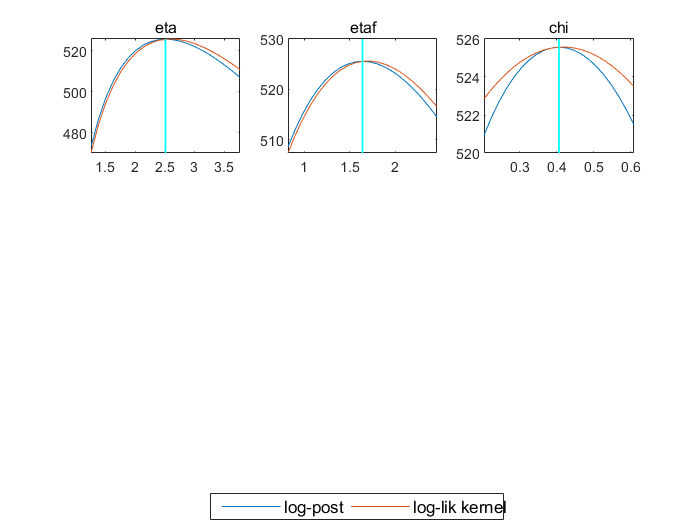


Fig 4. Mode checks under Rule 1.


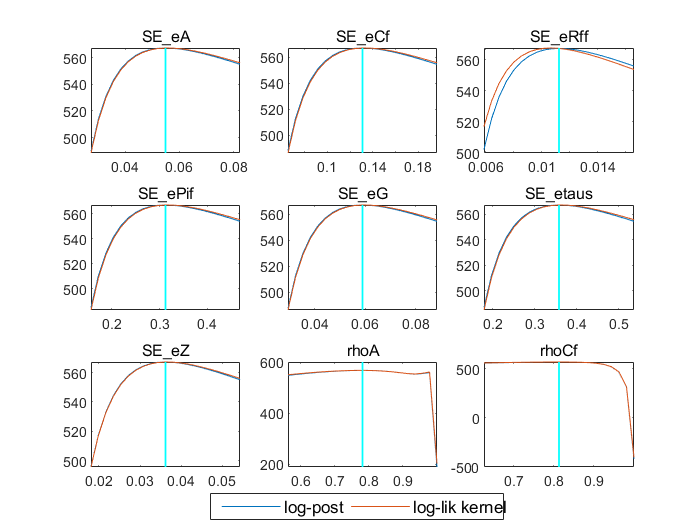

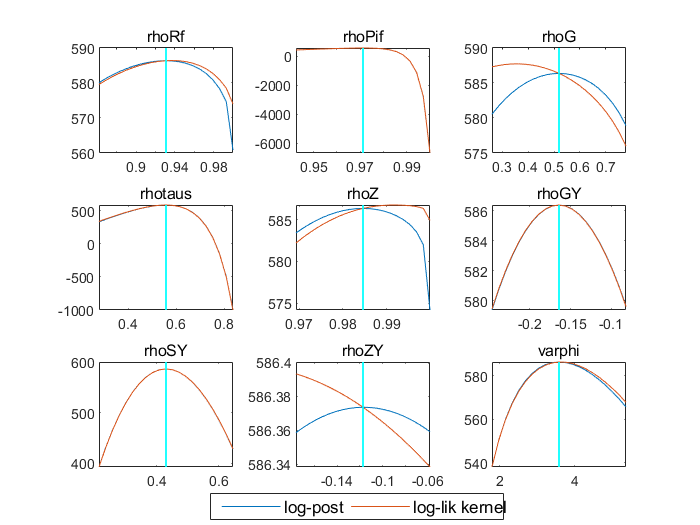

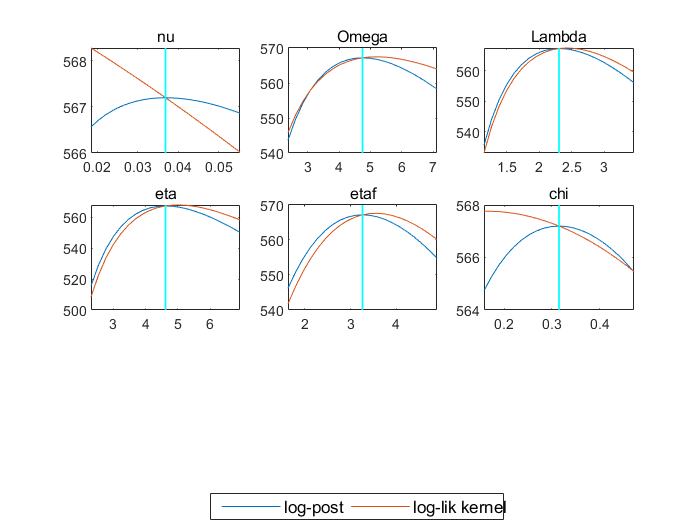


Fig 5. Mode checks under Rule 2.


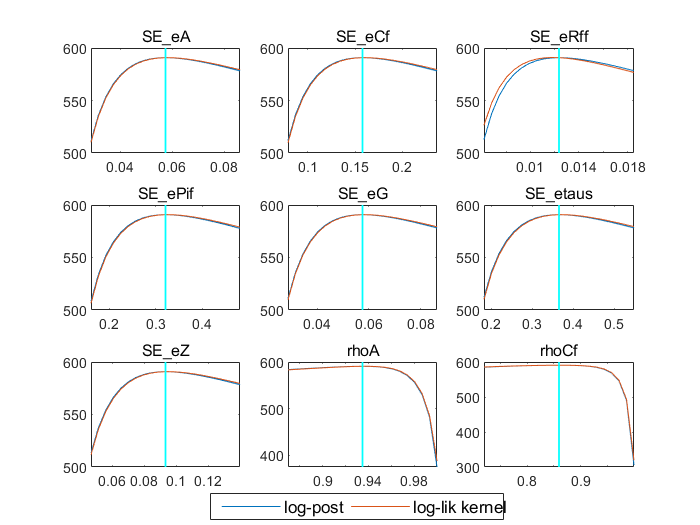

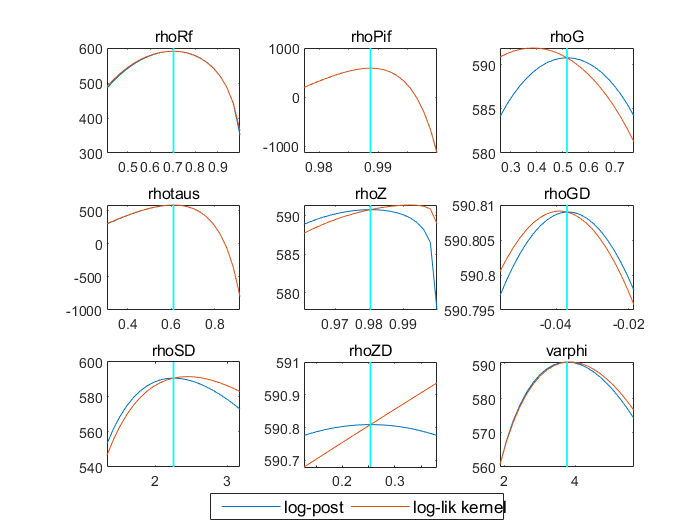

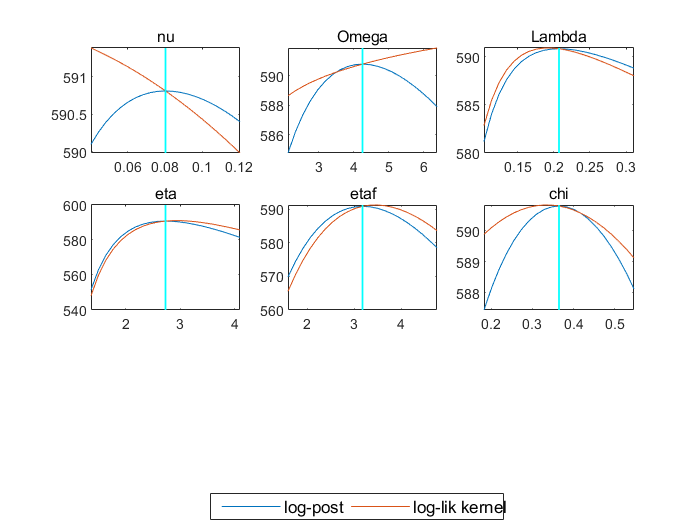


Fig 6. Mode checks under Rule 3.


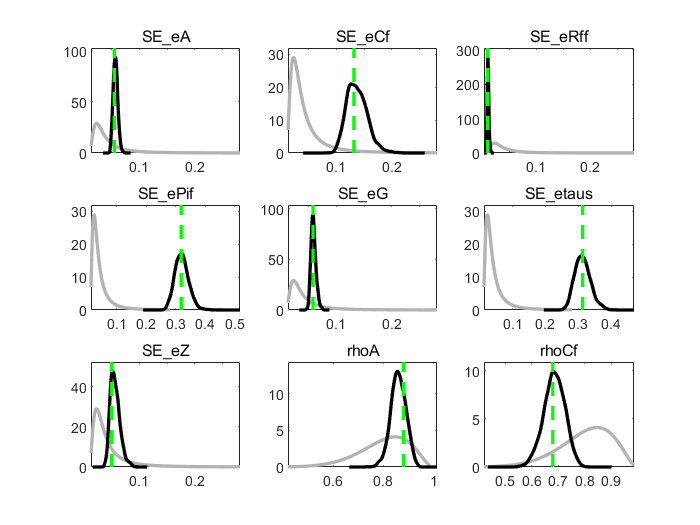

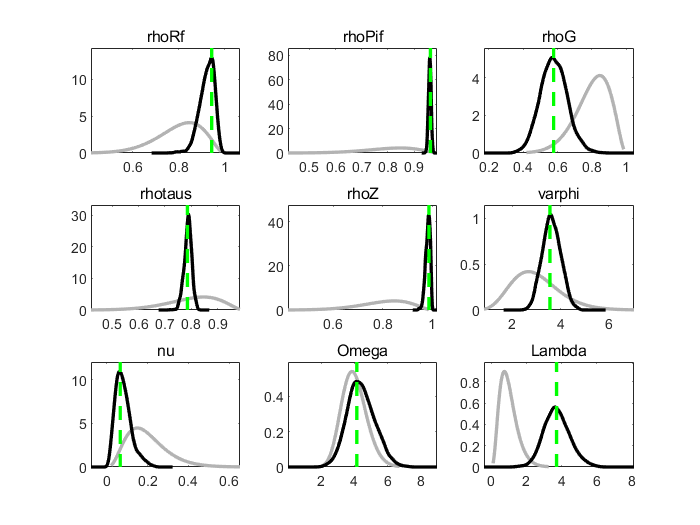

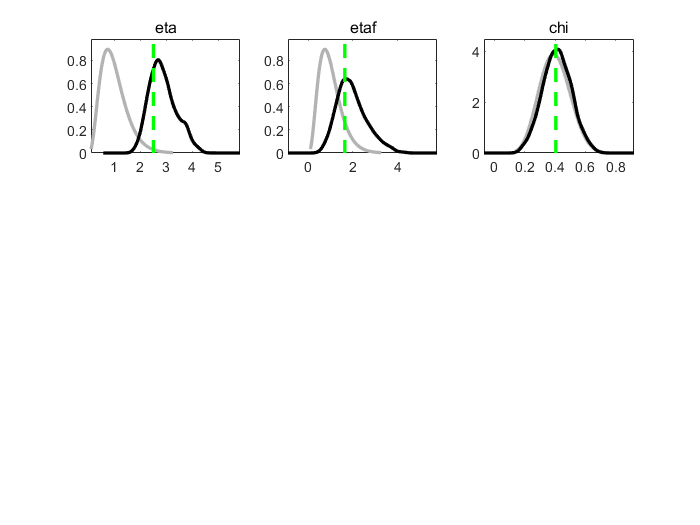


Fig 7. Prior and posterior distributions of parameters under Rule 1.


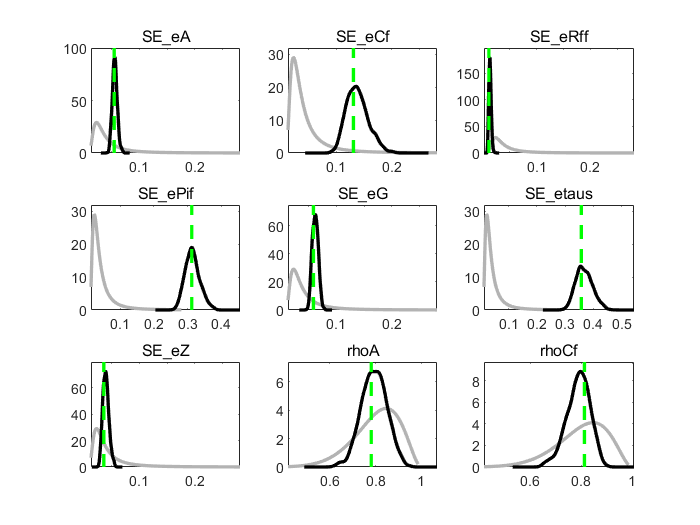

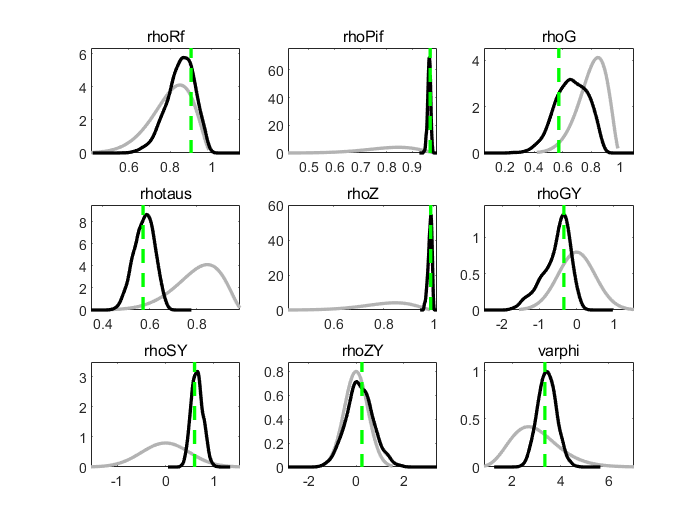

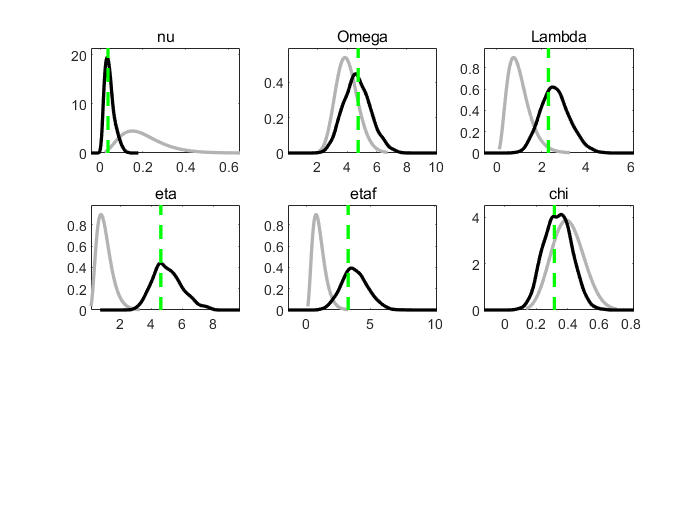


Fig 8. Prior and posterior distributions of parameters under Rule 2.


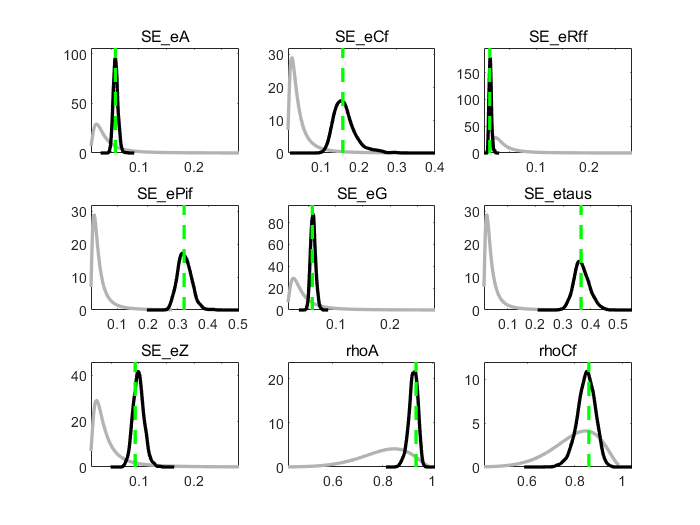

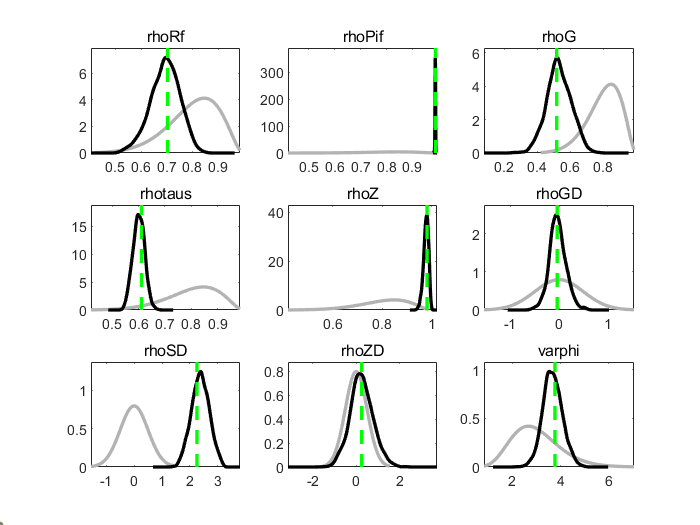

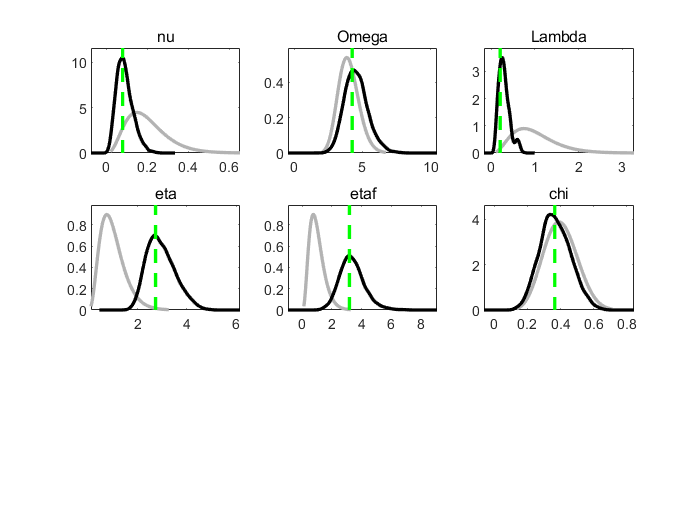


Fig 9. Prior and posterior distributions of parameters under Rule 3.
