# Supplementary material for: Land Finance and fiscal rules: An estimated DSGE model for Hong Kong
Source: PLoS One. 2026 Apr 15;21(4):e0346966. doi: 10.1371/journal.pone.0346966 (PMC13082663; doi:10.1371/journal.pone.0346966)
Supplement: S3 Table — (DOCX) [file pone.0346966.s003.docx]

Table 1. Posterior distributions when standard deviation of prior distribution is expanded to 1.1 times.

| parameter | Posterior distribution | | | | | |
| --- | --- | --- | --- | --- | --- | --- |
|  | Rule 1 | | Rule 2 | | Rule 3 | |
|  | Mean | 90% Interval | Mean | 90% Interval | Mean | 90% Interval |
| $\text{ν}$ | 0.0735 | [0.0145,0.1239] | 0.0384 | [0.0060,0.0711] | 0.0907 | [0.0172,0.1589] |
| $\text{Ω}$ | 4.4035 | [2.7512,5.9228] | 4.6992 | [3.0565,6.2239] | 4.6537 | [2.9593,6.1059] |
| $\text{λ}$ | 4.1100 | [2.8684,5.4721] | 2.7923 | [1.5542,3.9687] | 0.3008 | [0.0824,0.5113] |
| $\text{φ}$ | 3.6262 | [3.0372,4.2957] | 3.3979 | [2.7158,4.1025] | 3.7539 | [3.0672,4.4148] |
| $\text{η}$ | 2.9362 | [2.0162,3.8062] | 5.2503 | [3.7653,6.8236] | 3.1309 | [2.0181,4.1437] |
| $\text{η}_{\text{f}}$ | 2.0497 | [0.9412,3.1669] | 4.1119 | [2.2935,5.8663] | 3.8577 | [2.3022,5.3588] |
| $\text{χ}$ | 0.4197 | [0.2519,0.5900] | 0.3359 | [0.1830,0.4908] | 0.3707 | [0.1915,0.5244] |
| $\text{ρ}_{\text{a}}$ | 0.8583 | [0.8097,0.9071] | 0.7911 | [0.6682,0.8998] | 0.9220 | [0.8889,0.9534] |
| $\text{ρ}_{\text{c}_{\text{f}}}$ | 0.6695 | [0.5993,0.7424] | 0.7875 | [0.7154,0.8642] | 0.8397 | [0.7786,0.9088] |
| $\text{ρ}_{\text{p}_{\text{f}}}$ | 0.9568 | [0.9482,0.9663] | 0.9627 | [0.9518,0.9726] | 0.9878 | [0.9858,0.9895] |
| $\text{ρ}_{\text{r}_{\text{f}}}$ | 0.9286 | [0.8820,0.9785] | 0.8323 | [0.7009,0.9687] | 0.6830 | [0.5867,0.7841] |
| $\text{ρ}_{\text{τ}_{\text{s}}}$ | 0.7818 | [0.7577,0.8106] | 0.5635 | [0.5042,0.6319] | 0.5149 | [0.3853,0.6439] |
| $\text{ρ}_{\text{g}}$ | 0.5699 | [0.4201,0.7044] | 0.6547 | [0.4657,0.8290] | 0.5935 | [0.5539,0.6299] |
| $\text{ρ}_{\text{z}}$ | 0.9833 | [0.9709,0.9968] | 0.9854 | [0.9740,0.9973] | 0.9769 | [0.9610,0.9949] |
| $\text{ρ}_{\text{sy}}$ | - | - | 0.6890 | [0.4986,0.8710] | - | - |
| $\text{ρ}_{\text{gy}}$ | - | - | -0.5969 | [-1.2235,-0.0902] | - | - |
| $\text{ρ}_{\text{zy}}$ | - | - | 0.2386 | [-0.7419,1.2421] | - | - |
| $\text{ρ}_{\text{sd}}$ | - | - | - | - | 2.5531 | [1.9804,3.1333] |
| $\text{ρ}_{\text{gd}}$ | - | - | - | - | -0.0319 | [-0.3589,0.2478] |
| $\text{ρ}_{\text{zd}}$ | - | - | - | - | 0.3260 | [-0.6217,1.2550] |
| $\text{σ}_{\text{a}}$ | 0.0567 | [0.0493,0.0640] | 0.0559 | [0.0495,0.0630] | 0.0583 | [0.0509,0.0660] |
| $\text{σ}_{\text{c}_{\text{f}}}$ | 0.1389 | [0.1062,0.1697] | 0.1419 | [0.1073,0.1764] | 0.1696 | [0.1217,0.2137] |
| $\text{σ}_{\text{p}_{\text{f}}}$ | 0.3238 | [0.2844,0.3634] | 0.3179 | [0.2759,0.3561] | 0.0136 | [0.0096,0.0175] |
| $\text{σ}_{\text{r}_{\text{f}}}$ | 0.0116 | [0.0091,0.0141] | 0.0142 | [0.0101,0.0182] | 0.3250 | [0.2878,0.3603] |
| $\text{σ}_{\text{τ}_{\text{s}}}$ | 0.3155 | [0.2751,0.3526] | 0.3636 | [0.3143,0.4103] | 0.0587 | [0.0522,0.0655] |
| $\text{σ}_{\text{g}}$ | 0.0587 | [0.0516,0.0659] | 0.0631 | [0.0529,0.0717] | 0.3666 | [0.3203,0.4128] |
| $\text{σ}_{\text{z}}$ | 0.0542 | [0.0421,0.0665] | 0.0383 | [0.0301,0.0459] | 0.1023 | [0.0833,0.1187] |

Table 2. Posterior distributions estimated using subsamples.

| parameter | Posterior distribution | | | | | |
| --- | --- | --- | --- | --- | --- | --- |
|  | Rule 1 | | Rule 2 | | Rule 3 | |
|  | Mean | 90% Interval | Mean | 90% Interval | Mean | 90% Interval |
| $\text{ν}$ | 0.0889 | [0.0201,0.1468] | 0.0612 | [0.0135,0.1258] | 0.0945 | [0.0243,0.1500] |
| $\text{Ω}$ | 4.1937 | [2.9319,5.4075] | 4.3064 | [3.0836,5.5343] | 4.4004 | [3.2574,5.7239] |
| $\text{λ}$ | 3.9423 | [2.7535,4.9994] | 3.1368 | [1.9969,4.1412] | 0.4634 | [0.1667,0.7734] |
| $\text{φ}$ | 3.5640 | [2.9147,4.1817] | 3.0527 | [2.2377,3.6805] | 3.7200 | [3.0497,4.2785] |
| $\text{η}$ | 2.2991 | [1.6585,2.9106] | 3.9976 | [2.7702,5.1528] | 2.6009 | [1.9228,3.2534] |
| $\text{η}_{\text{f}}$ | 2.1112 | [1.1532,3.0570] | 3.8668 | [2.3112,5.4149] | 3.2327 | [2.2882,4.0502] |
| $\text{χ}$ | 0.4557 | [0.2833,0.6113] | 0.3498 | [0.1931,0.5040] | 0.4044 | [0.2483,0.5306] |
| $\text{ρ}_{\text{a}}$ | 0.8712 | [0.8201,0.9145] | 0.9385 | [0.8562,0.9915] | 0.9184 | [0.8903,0.9565] |
| $\text{ρ}_{\text{c}_{\text{f}}}$ | 0.6405 | [0.5624,0.7248] | 0.6825 | [0.5871,0.7889] | 0.8211 | [0.7575,0.8840] |
| $\text{ρ}_{\text{p}_{\text{f}}}$ | 0.9589 | [0.9505,0.9662] | 0.9667 | [0.9581,0.9775] | 0.9878 | [0.9862,0.9896] |
| $\text{ρ}_{\text{r}_{\text{f}}}$ | 0.9480 | [0.9085,0.9862] | 0.9206 | [0.8625,0.9871] | 0.7382 | [0.6348,0.7812] |
| $\text{ρ}_{\text{τ}_{\text{s}}}$ | 0.7763 | [0.7484,0.8018] | 0.6191 | [0.5516,0.6693] | 0.6145 | [0.5668,0.6530] |
| $\text{ρ}_{\text{g}}$ | 0.5848 | [0.4392,0.7267] | 0.6066 | [0.4741,0.7786] | 0.5157 | [0.3813,0.6574] |
| $\text{ρ}_{\text{z}}$ | 0.9760 | [0.9562,0.9942] | 0.9829 | [0.9712,0.9951] | 0.9740 | [0.9557,0.9935] |
| $\text{ρ}_{\text{sy}}$ | - | - | 0.5512 | [0.4063,0.7395] | - | - |
| $\text{ρ}_{\text{gy}}$ | - | - | -0.4196 | [-0.8206,-0.0567] | - | - |
| $\text{ρ}_{\text{zy}}$ | - | - | -0.0589 | [-0.8468,0.9129] | - | - |
| $\text{ρ}_{\text{sd}}$ | - | - | - | - | 2.1548 | [1.6979,2.5820] |
| $\text{ρ}_{\text{gd}}$ | - | - | - | - | -0.1123 | [-0.4071,0.1454] |
| $\text{ρ}_{\text{zd}}$ | - | - | - | - | 0.3263 | [-0.5055,1.2009] |
| $\text{σ}_{\text{a}}$ | 0.0569 | [0.0495,0.0640] | 0.0566 | [0.0502,0.0633] | 0.0602 | [0.0519,0.0688] |
| $\text{σ}_{\text{c}_{\text{f}}}$ | 0.1489 | [0.1078,0.1865] | 0.1512 | [0.1109,0.1871] | 0.1640 | [0.1293,0.1999] |
| $\text{σ}_{\text{p}_{\text{f}}}$ | 0.3331 | [0.2931,0.3739] | 0.3212 | [0.2832,0.3623] | 0.3325 | [0.2872,0.3643] |
| $\text{σ}_{\text{r}_{\text{f}}}$ | 0.0118 | [0.0093,0.0144] | 0.0118 | [0.0093,0.0139] | 0.0130 | [0.0101,0.0166] |
| $\text{σ}_{\text{τ}_{\text{s}}}$ | 0.3293 | [0.2862,0.3685] | 0.3633 | [0.3166,0.4267] | 0.3796 | [0.3346,0.4241] |
| $\text{σ}_{\text{g}}$ | 0.0596 | [0.0516,0.0675] | 0.0618 | [0.0530,0.0681] | 0.0596 | [0.0516,0.0676] |
| $\text{σ}_{\text{z}}$ | 0.0525 | [0.0388,0.0676] | 0.0336 | [0.0231,0.0413] | 0.0916 | [0.0747,0.1080] |
